# Microbiome of pear psyllids: A tale about closely related species sharing their endosymbionts

**DOI:** 10.1111/1462-2920.16180

**Published:** 2022-09-09

**Authors:** Liliya Štarhová Serbina, Domagoj Gajski, Barbora Pafčo, Ludek Zurek, Igor Malenovský, Eva Nováková, Hannes Schuler, Jessica Dittmer

**Affiliations:** ^1^ Faculty of Science and Technology Free University of Bozen‐Bolzano Bolzano Italy; ^2^ Department of Botany and Zoology, Faculty of Science Masaryk University Brno Czech Republic; ^3^ Institute of Vertebrate Biology Czech Academy of Sciences Brno Czech Republic; ^4^ Central European Institute of Technology University of Veterinary and Pharmaceutical Sciences Brno Czech Republic; ^5^ Department of Microbiology, Nutrition and Dietetics/CINeZ Czech University of Life Sciences Prague Czech Republic; ^6^ Faculty of Science University of South Bohemia Ceske Budejovice Czech Republic; ^7^ Competence Centre for Plant Health Free University of Bozen‐Bolzano Bolzano Italy; ^8^ Université d'Angers, Institut Agro INRAE, IRHS, SFR Quasav Angers France

## Abstract

Psyllids are phloem‐feeding insects that can transmit plant pathogens such as phytoplasmas, intracellular bacteria causing numerous plant diseases worldwide. Their microbiomes are essential for insect physiology and may also influence the capacity of vectors to transmit pathogens. Using 16S rRNA gene metabarcoding, we compared the microbiomes of three sympatric psyllid species associated with pear trees in Central Europe. All three species are able to transmit ‘*Candidatus* Phytoplasma pyri’, albeit with different efficiencies. Our results revealed potential relationships between insect biology and microbiome composition that varied during psyllid ontogeny and between generations in *Cacopsylla pyri* and *C. pyricola*, as well as between localities in *C. pyri*. In contrast, no variations related to psyllid life cycle and geography were detected in *C. pyrisuga*. In addition to the primary endosymbiont *Carsonella ruddii*, we detected another highly abundant endosymbiont (unclassified Enterobacteriaceae). *C. pyri* and *C. pyricola* shared the same taxon of Enterobacteriaceae which is related to endosymbionts harboured by other psyllid species from various families. In contrast, *C. pyrisuga* carried a different Enterobacteriaceae taxon related to the genus *Sodalis*. Our study provides new insights into host–symbiont interactions in psyllids and highlights the importance of host biology and geography in shaping microbiome structure.

## 1. INTRODUCTION

Insects establish numerous associations with viruses, bacteria and fungi and can act as vectors of pathogens that cause severe diseases of humans and plants. In particular, some hemipteran insects are devastating pests of annual and perennial crops, mainly due to their ability to transmit numerous phytopathogenic bacteria and viruses, causing serious diseases and leading to significant agricultural losses (Alma et al., 2008; Wilkinson, 2012). The vector competence, that is, the ability of an insect to transmit pathogens, depends on the biology of both the insect vector and the pathogen, as well as on their interaction (Gonella et al., 2019; Orlovskis et al., 2015; Perilla‐Henao & Casteel, 2016; Tamborindeguy et al., 2017). Despite rapid progress in insect‐borne pathogen research in the last decade, it is still poorly understood which factors could favour or impede pathogen transmission.

Recent studies on insect microbiomes have revealed the essential role of the microbiome in the development, physiology and evolution of their hosts (Brucker & Bordenstein, 2013; Newell & Douglas, 2014; Shin et al., 2011; Storelli et al., 2011; van Opstal & Bordenstein, 2019). Insect symbionts are known to provide their hosts with important benefits (synthesizing essential amino acids and vitamins, or protection against predators and pathogens) (Douglas, 2015; Hypša & Nováková, 2008; Jaenike, 2012; Moran et al., 2008; Wilkinson, 2012) but can also manipulate their host's reproduction or shorten its lifespan to spread and persist within host populations (Engelstädter & Hurst, 2009; Hurst & Frost, 2015; Macke et al., 2017; McCutcheon et al., 2019; McMeniman et al., 2009). Moreover, some insect symbionts are known to affect the host's ability to transmit pathogens, which may have a direct impact on the dynamics of vector‐borne diseases (Bourtzis et al., 2014; Chu et al., 2016; Fagen et al., 2012; Gonella et al., 2019; Gottlieb et al., 2010; McMeniman et al., 2009). For instance, the presence of the bacterial endosymbiont *Hamiltonella* in the whitefly *Bemisia tabaci* (Hemiptera: Aleyrodidae) was shown to increase the acquisition and transmission efficiency of the Tomato yellow leaf curl virus (TYLCV) in this vector species (Gottlieb et al., 2010; Su et al., 2013). Similarly, *Wolbachia* may mitigate or impede pathogen acquisition and transmission in the Asian citrus psyllid *Diaphorina citri* (Hemiptera: Liviidae) (Fagen et al., 2012; Hosseinzadeh et al., 2019; Kruse et al., 2017; Song et al., 2019). Furthermore, *Wolbachia* was shown to inhibit the transmission of a plant virus by *Nilaparvata lugens* (Hemiptera: Delphacidae) (Gong et al., 2020). These observations indicate that certain members of insect microbiomes can have a strong impact on vector‐borne disease transmission.

The composition of an insect‐associated bacterial community can vary along an insect's life cycle and shift in response to changes in environmental conditions and/or insect biology (Colman et al., 2012; Fromont et al., 2017; Macke et al., 2017; Meng et al., 2019; Nováková et al., 2017; Yun et al., 2014). For instance, a shift in abundant bacterial taxa was observed across different life stages of *D. citri* (Meng et al., 2019). Likewise, seasonal changes affected microbial abundance and microbiome composition in mosquitoes and fleas (Cohen et al., 2015; Nováková et al., 2017). Furthermore, prolonged larval diapause in the parasitoid wasp *Nasonia vitripennis* modified microbiome composition in adults after termination of diapause (Dittmer & Brucker, 2021). Notably, *Wolbachia* does not survive prolonged larval diapause in this species (Perrot‐Minnot et al., 1996). This variability could potentially affect the vector competence of an insect host (Duguma et al., 2015; Ramsey et al., 2017; Rodríguez‐Ruano et al., 2018), for instance, when a bacterial antagonist of the vectored pathogen is lost from the microbiome due to insect ontogeny or changes in environmental conditions.

**FIGURE 1:**
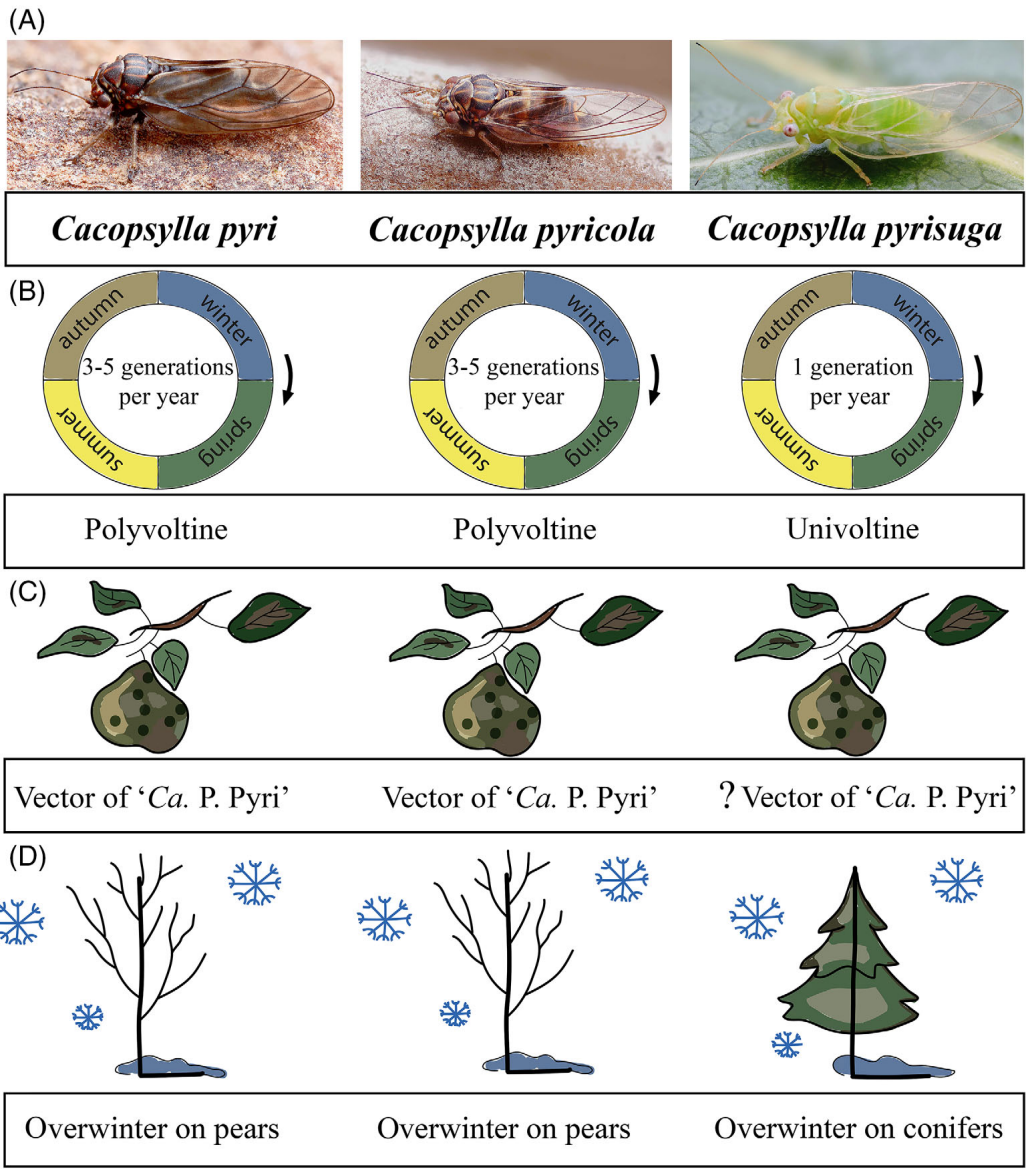
Schematic overview of the biology of the three pear‐associated *Cacopsylla* species. (**A**) Adult individuals, (**B**) differences in voltinism (generations per year), (**C**) vector competence, and (**D**) differences in overwintering strategies. The vector competence of *Cacopsylla pyrisuga* is marked with a question mark (?) because its vector capacity has been demonstrated for Austrian populations but is uncertain for CZ.

Psyllids (Insecta: Hemiptera: Psylloidea) are among the most economically important vectors of bacterial pathogens (Jing et al., 2014; Wilkinson, 2012). Among the major psyllid vectors, *D. citri* and *Bactericera cockerelli* (Triozidae) transmit ‘*Candidatus* Liberibacter’, which causes Huanglongbing (citrus greening) and zebra chip disease of potato (Bové, 2006; Hall et al., 2012; Hansen et al., 2008). In addition, psyllids transmit various strains of ‘*Candidatus* Phytoplasma’, intracellular bacterial pathogens which cause diseases of fruit trees with high economic importance in Europe and worldwide, such as apple proliferation on apples, stone fruit yellows on apricots and plums and Pear Decline on pears and peaches (Jarausch et al., 2019). The psyllid vectors of phytoplasmas comprise several Palearctic species from the genus *Cacopsylla* (Psyllidae) (Jarausch et al., 2019; Tamborindeguy et al., 2017). Among them, *Cacopsylla pyri*, *C. pyricola* and *C. pyrisuga* are sympatric in Central Europe and represent the most common psyllid species associated with pear trees. *Cacopsylla pyri* and *C. pyricola* are important vectors of ‘*Candidatus* Phytoplasma pyri causing Pear Decline and thus belong to the most harmful pests of pears in Europe and North America where they were introduced (Jarausch et al., 2019; Tougeron et al., 2021). Both vectors produce up to 3–5 generations per year and show resistance to insecticides, making it difficult to control population outbreaks (Civolani, 2012). In contrast to *C. pyri* and *C. pyricola*, *C. pyrisuga* had been considered a non‐vector species until recently, when its vector competence was experimentally proven in Austria (Riedle‐Bauer et al., 2022). However, it is not known to what extent this species acts as a vector in other parts of Europe. Importantly, *C. pyri* and *C. pyricola* differ from *C. pyrisuga* in numerous aspects of their biology, that is, in their life cycles, voltinism and feeding behaviour (Figure 1). *C. pyri* and *C. pyricola* represent closely related, seasonally polymorphic, polyvoltine species (Cho et al., 2020; Conci et al., 1993; Hodkinson, 2009; Soroker et al., 2013). They complete their life cycles on their host‐plants (*Pyrus* spp.), where they also predominantly overwinter (Hodkinson, 2009; Lauterer, 1999) (Figure 1). Interestingly, in its invasive range in North America, where *C. pyricola* was introduced from Europe, a number of individuals were found overwintering on conifers or other shelter plants (e.g., in apple orchards), while only a fraction of the population was associated solely with pear trees during an entire year (Horton et al., 1992, 1994). This suggests that *C. pyricola* individuals may overwinter on conifers in North America, but this association does not appear to be obligate. Both *C. pyri* and *C. pyricola* species produce several long‐day summer generations (with small‐sized, light‐coloured morphs) and usually one morphologically different short‐day overwintering generation (with a large‐sized, dark‐coloured morph) (Lauterer, 1999; Soroker et al., 2013; Figure S1). A new annual cycle begins in spring when the overwintered adults lay eggs, which give rise to the first summer generation (Figure S2A) (Hodkinson, 2009). In central Europe, the overwintering generation starts at the beginning of autumn with the eggs laid by adults of the last summer‐form generation. Later in autumn, the adults from the overwintering generation undergo a reproductive diapause which ends in the middle of winter, followed by a very slow reproductive development, and in spring a new annual cycle starts over (Figure S2A) (Hodkinson, 2009; Horton et al., 1998; Lauterer, 1999). In contrast, *C. pyrisuga* (Figures 1 and S1) produces only a single generation per year. It is also monophagous on pear trees (i.e., its immatures cannot develop on any other plant taxa), but in contrast to *C. pyri* and *C. pyricola*, this species has an obligate overwintering association with conifers (Figure 1) where it undergoes a reproductive diapause as adults (Hodkinson, 2009). In spring, overwintered adults of *C. pyrisuga* move back to pear trees where they lay their eggs (Figure S2B) (Lauterer, 1999). In summer, young adults migrate to shelters on occasional plants and afterwards on conifers. In light of the differences in host biology and possibly also vector competence (Figure 1), *C. pyri*, *C. pyricola* and *C. pyrisuga* represent an interesting study system to investigate the potential links between host biology, vector competence and the microbiome.

Like other groups of sap‐feeding insects, psyllids have established obligatory relationships with a primary endosymbiont which supplies the insect with essential amino acids lacking from its plant‐sap diet. The primary endosymbiont of psyllids is ‘*Candidatus* Carsonella ruddii’ (hereafter *Carsonella*), which is present in all psyllid species studied to date (e.g., Cooper et al., 2017; Cooper, Garczynski, & Horton, 2015; Morrow et al., 2017; Overholt et al., 2015; Song et al., 2019; Thao et al., 2000). *Carsonella* is localized in specialized host cells, bacteriocytes, which constitute a tissular structure called a bacteriome. In addition, numerous other secondary endosymbionts were detected in various psyllids. Depending on the psyllid species, these belong to variable bacterial groups, notably Alphaproteobacteria (*Wolbachia*), Betaproteobacteria (*Profftella*) and Enterobacteriaceae (*Arsenophonus*, *Sodalis*, unclassified Enterobacteriaceae) (Fromont et al., 2016, 2017; Fu et al., 2020; Morrow et al., 2017, 2020; Nakabachi et al., 2013; Nakabachi, Malenovský, et al., 2020; Nakabachi, Piel, et al., 2020). Some of these symbionts can be found in various host tissues, including the bacteriome, fat body, hemolymph, salivary glands and reproductive organs (Pontes et al., 2008; Wilkinson, 2012).

The aim of this work was to investigate and compare the microbiomes of the three psyllid species sympatric on pear trees in Central Europe, *C. pyri*, *C. pyricola* and *C. pyrisuga*. Our specific aims were to (i) characterize the microbiomes of various populations of *C. pyri*, *C. pyricola* and *C. pyrisuga*, (ii) investigate potential differences in microbiome composition between the three pear psyllid species, and (iii) investigate the impact of host ontogeny and seasonal generations on psyllid microbiome. To this end, our dataset comprises several populations from various geographical localities across different life stages and different generations for the polyvoltine species. Our results provide insights into the potential effects of host biology and geography on the microbiomes of pear psyllids.

## 2. MATERIALS AND METHODS

### 2.1. Sample collection

Most individuals of *Cacopsylla pyri*, *C. pyricola* and *C. pyrisuga* were selected from samples collected throughout an entire year from February 2020 until February 2021 in a pear orchard in Starý Lískovec (Brno, Czech Republic) (Tables 1 and S1; Figure S2). The sampling effort in this orchard was standardized for the collection of adults, with a time interval of 7–10 days between each visit. At each visit, psyllids were collected from five branches of five randomly chosen pear trees. Adults were collected using entomological sweeping nets and a beating tray, while eggs and 4–5th instar immatures were collected with a camelhair brush. Adults of *C. pyricola* were selected for sequencing based on the abundance peaks during the sampling year, representing different generations of this species (Figure S2A). In contrast to *C. pyricola*, the population of *C. pyri* did not show clear seasonal peaks but specimens were present at a constant but low density throughout the sampling year. Therefore, specimens of *C. pyri* likely representing different generations were selected for sequencing based on the information on its life cycle published for the sampling region (Lauterer, 1999). Regarding the univoltine *C. pyrisuga*, specimens were selected for sequencing from its only seasonal peak in late spring and early summer (Figure S2B). We also included *C. pyrisuga* specimens collected at the beginning of spring, corresponding to adults from the previous year that had overwintered on conifers and migrated back to pear trees in early spring (remigrants). In addition to Starý Lískovec, specimens of *C. pyri* were also collected in two other localities in the Czech Republic and one locality in Italy, specimens of *C. pyricola* in three other localities in the Czech Republic and one locality in France, and additional specimens of *C. pyrisuga* were obtained in another locality in the Czech Republic (Tables 1 and 1S). There is no information on the phytoplasma infection status of the pear orchards where we collected psyllids. Psyllids were identified to species based on adult and immature morphology (Burckhardt, 2010; Ossiannilsson, 1992).

**TABLE 1:** List of analysed psyllid samples with information on their developmental stage, sex, number of pooled specimens (N), sampling and locality data with abbreviations used in the figures. CZ = Czech Republic

Psyllid species	Developmental stage, gender	N samples	Date	Locality with abbreviations	Latitude	Longitude
*Cacopsylla pyri*	Adult, ♂	5	04 December 2019	CZ, Litenčice = **CZ1**	−49.2056	−17.2131
	Adult, ♀	5	04 December 2019	CZ, Litenčice = **CZ1**	−49.2056	−17.2131
	Immature	5	04 December 2019	CZ, Litenčice = **CZ1**	−49.2056	−17.2131
	Adult, ♂	1	15 February 2020	CZ, Starý Lískovec (Brno) = **CZ2**	−49.1623	−16.5868
	Adult, ♀	1	15 February 2020	CZ, Starý Lískovec (Brno) = **CZ2**	−49.1623	−16.5868
	Immature	2	17 May 2020	CZ, Starý Lískovec (Brno) = **CZ2**	−49.1623	−16.5868
	Adult, ♂	2	07 July 2020	CZ, Starý Lískovec (Brno) = **CZ2**	−49.1623	−16.5868
	Adult, ♀	2	07 July 2020	CZ, Starý Lískovec (Brno) = **CZ2**	−49.1623	−16.5868
	Immature	5	07 July 2020	CZ, Staré Město (Svitavy) = **CZ3**	−49.7857	−16.6882
	Adult, ♂	4	08 May 2020	Italy, Merano, Plaus = **IT**	−46.6545	−11.0421
	Adult, ♀	4	08 May 2020	Italy, Merano, Plaus = **IT**	−46.6545	−11.0421
*Cacopsylla pyricola*	Adult, ♂	5	23 February 2020	CZ, Starý Lískovec (Brno) = **CZ2**	−49.1623	−16.5868
	Adult, ♀	5	23 February 2020	CZ, Starý Lískovec (Brno) = **CZ2**	−49.1623	−16.5868
	Adult, ♂	5	10 May 2020	CZ, Starý Lískovec (Brno) = **CZ2**	−49.1623	−16.5868
	Adult, ♀	5	10 May 2020	CZ, Starý Lískovec (Brno) = **CZ2**	−49.1623	−16.5868
	Immature	5	10 May 2020	CZ, Starý Lískovec (Brno) = **CZ2**	−49.1623	−16.5868
	Adult, ♂	5	02 August 2020	CZ, Starý Lískovec (Brno) = **CZ2**	−49.1623	−16.5868
	Adult, ♀	5	02 August 2020	CZ, Starý Lískovec (Brno) = **CZ2**	−49.1623	−16.5868
	Immature	3	13 September 2020	CZ, Starý Lískovec (Brno) = **CZ2**	−49.1623	−16.5868
	Immature	2	27 September 2020	CZ, Starý Lískovec (Brno) = **CZ2**	−49.1623	−16.5868
	Adult, ♂	4	18 October 2020	CZ, Starý Lískovec (Brno) = **CZ2**	−49.1623	−16.5868
	Adult, ♀	4	18 October 2020	CZ, Starý Lískovec (Brno) = **CZ2**	−49.1623	−16.5868
	Adult, ♂	4	06 July 2020	CZ, Božanov = **CZ4**		
	Adult, ♀	5	06 July 2020	CZ, Božanov = **CZ4**		
	Adult, ♂	5	12 July 2020	CZ, Hradec Králové = **CZ5**		
	Adult, ♀	5	12 July 2020	CZ, Hradec Králové = **CZ5**		
	Adult, ♂	4	14 August2020	CZ, Staré Hutě = **CZ6**	−49.1394	−17.2867
	Adult, ♀	3	14 August 2020	CZ, Staré Hutě = **CZ6**	−49.1394	−17.2867
	Adult, ♂	2	01 August 2018	France, Haut‐Rhin, Wittelsheim Rothmoos = **FR**	−47.7782	−7.2386
	Adult, ♀	3	01 August 2018	France, Haut‐Rhin, Wittelsheim Rothmoos = **FR**	−47.7782	−7.2386
*Cacopsylla pyrisuga*	Adult, ♂	3	29 March 2020	CZ, Starý Lískovec (Brno) = **CZ2**	−49.1623	−16.5868
	Adult, ♀	5	29 March 2020	CZ, Starý Lískovec (Brno) = **CZ2**	−49.1623	−16.5868
	Immature	5	10 May 2020	CZ, Starý Lískovec (Brno) = **CZ2**	−49.1623	−16.5868
	Adult, ♂	3	02 June 2020	CZ, Starý Lískovec (Brno) = **CZ2**	−49.1623	−16.5868
	Adult, ♀	5	02 June 2020	CZ, Starý Lískovec (Brno) = **CZ2**	−49.1623	−16.5868
	Adult, ♂	5	09 May 2020	CZ, Staré Město (Svitavy) = **CZ3**	−46.5000	−11.3626
	Adult, ♀	5	09 May 2020	CZ, Staré Město (Svitavy) = **CZ3**	−46.5000	−11.3626
	Immature	5	09 May 2020	CZ, Staré Město (Svitavy) = **CZ3**	−46.5000	−11.3626
	Egg	3	09 May 2020	CZ, Staré Město (Svitavy) = **CZ3**	−46.5000	−11.3626

### 2.2. DNA extraction and 16S rRNA gene amplicon sequencing

DNA was extracted from 151 psyllid individuals (*C. pyri* [*N* = 36], *C. pyricola* [*N* = 79], *C. pyrisuga* [*N* = 39]), as well as three egg samples (5 egg specimens per sample) from *C. pyrisuga* (Table S1) using E.Z.N.A.® Tissue DNA Kit (Omega Bio‐tek). The specimens represented three categories of psyllid life stages: adults (*N* = 119), immatures (*N* = 32) and eggs (*N* = 3). Two‐step‐PCR amplification was performed using KAPA2G Robust HotStart Polymerase (Merck) (Pafčo et al., 2018). In the first step, the V3–V4 variable region of the 16S rRNA gene was targeted using primer pair 341F and 805F (Klindworth et al., 2013) containing ‘tails’ serving as priming sites for the second PCR. The thermal conditions were as follows: 95°C for 3 min, 30 cycles (95°C for 15 s, 55.5°C for 15 s, and 72°C for 15 s) and 72°C for 3 min. In the second step, Nextera primers with sample‐specific barcodes and sequencing adaptors were used under the following conditions: 95°C for 3 min, 16 cycles (95°C for 15 s, 55°C for 30 s, and 72°C for 30 s), and 72°C for 3 min. Each sample was prepared in duplicate with a unique barcode to minimize the presence of putative chimeras or other kinds of non‐biological variants (Pafčo et al., 2018). Six negative controls were also included from extraction to sequencing, which was performed on an Illumina MiSeq platform using MiSeq Reagent Kit v3 (2 × 300 bp) chemistry.

Five immature specimens of *C. pyri* collected from the site CZ1 in winter (Table S1) were checked for parasitoid COI sequences due to their unusual microbiome composition (e.g., missing *Carsonella* and unclassified Enterobacteriaceae, Figure S4). PCR amplification was performed using DreamTaq PCR Master Mix (Thermo Fisher) and the primer pair LCO and HCO (Folmer et al., 1994) with the following thermal conditions: 35 cycles of 95°C for 30 s, 50°C for 30 s, and 72°C for 60 s. Parasitoid DNA was amplified from two out of the five individuals. The two amplified parasitoid COI sequences were identical and therefore only one sequence from a single specimen (P18) was submitted to GenBank with the accession number OK561501. Based on BLAST search, the obtained parasitoid sequences were identified as *Syrphophagus aphidivorus* (Hymenoptera: Encyrtidae) with 98.84% identity. Ten adult specimens of *C. pyri* were additionally checked for the presence of parasitoid DNA using two primer sets, first using the LCO and HCO primer set (Folmer et al., 1994) (the same we used earlier for the detection of parasitoid DNA in immatures), which did not produce any PCR products. Then, we used the *Cacopsylla*‐specific primer set VPm_COI_F2 and VPm_COI_R4 (Oettl & Schlink, 2015) and successfully amplified psyllid DNA from all tested individuals using following PCR conditions: 35 cycles of 95°C for 30 s, 46°C for 30 s, and 72°C for 60 s.

### 2.3. Microbiome analyses

The obtained reads were quality‐checked using FastQC (Andrews, 2010) and trimmed to a final length of 250 bp using Trimmomatic implemented on Galaxy (Afgan et al., 2018) to remove low‐quality bases at the ends of the reads. Paired reads were then joined using the *join_paired_ends.py* script from QIIME (v1.9) (Caporaso et al., 2010). The joined reads were denoized and assigned to ASVs using the dada2 plugin in QIIME2 (Bolyen et al., 2019). Taxonomy was assigned to the representative sequence of each ASV based on the Silva database (v132) (Yilmaz et al., 2014) using the RDP classifier (Table S2). ASVs identified as mitochondria and chloroplasts as well as low‐abundance ASVs with <3 reads were removed from the ASV table. All ASVs amplified in the negative controls (*N* = 193) were removed from the ASV table to eliminate potential contaminants from downstream analyses.

All statistical analyses and graphics were performed in QIIME (v1.9) and R (v3.6.3), using the following packages: vegan, MASS, ggplot2, edgeR and indicspecies. Bacterial species richness and diversity were determined using the species richness estimator Chao 1 and the Shannon index of diversity after rarefaction of all samples to an even sampling depth of 10,000 reads. Alpha diversity indices were compared (i) between host species, and (ii) within each species, between different developmental stages, seasons, localities and between males and females). This was done using pairwise *t*‐tests after 1000 Monte Carlo permutations, as implemented in the QIIME script *compare_alpha_diversity.py*. Global differences in beta diversity between the three psyllid species were analysed using Principal Coordinates Analysis (PCoA) based on Bray–Curtis distances and ANOSIM after 10,000 permutations using the QIIME script *compare_categories.py*. Finer scale differences in microbiome composition depending on psyllid host developmental stage, generations/seasonality, locality, and sex were investigated within each species using nonmetric multidimensional scaling (NMDS) based on Bray–Curtis distances. The EnvFit function from the R package ‘vegan’ was then applied to fit the host variables onto the NMDS ordination scores and to identify significant correlations between microbiome composition and host traits. For *C. pyri* and *C. pyricola*, development and seasonal generations were analysed both separately and together, due to their potential biological interplay. For *C. pyrisuga*, which produces only one generation per year, microbiome composition was compared between overwintered adults (remigrants) and adults of a new summer generation. To compare the microbiome composition between males and females, samples representing immatures and eggs, for which the sex could not be identified, were removed from the dataset. A Venn diagram indicating the amount of shared versus species‐specific ASVs was produced using the Bioconductor package ‘limma’ using the data from the only locality (CZ2) where all three *Cacopsylla* species were present at the adult stage.

Bacterial genera that were mainly responsible for the differences in microbiomes between the three psyllid species were determined using two different approaches: (i) The indval function from the R package ‘indicspecies’. This function performs Indicator Species Analysis by calculating an Indicator Value (IndVal) for each bacterial taxon (De Caceres & Legendre, 2009). The bacterial genera with a *p*‐value < 0.034 were considered significant. (ii) The R package ‘edgeR’ for differential gene expression analysis (Robinson et al., 2010). Bacterial abundance was based on log CPM (counts per million) values (Table S4). Only genera present in at least 15 samples were included in the analysis and bacterial genera were considered differentially abundant with a log2‐fold change ≥1 and with an FDR‐adjusted *p*‐value ≤ 0.05.

### 2.4. Phylogenetic reconstruction of unclassified Enterobacteriaceae endosymbionts

To understand the phylogenetic relationships of ASVs identified as unclassified Enterobacteriaceae between the three *Cacopsylla* species, a phylogenetic analysis of these ASVs was performed. This analysis included (1) the seven most dominant ASVs out of 10 found in *Cacopsylla pyri*, (2) all 20 ASVs found in *C. pyricola*, and (3) the 10 most dominant ASVs out of 44 found in *C. pyrisuga* (11,914–139,391 reads per ASV) (Table S2). The 16S rRNA gene sequences of several endosymbionts from other psyllid species, namely *C. myrthi* (AF263559), *C. burckhardti* (TAAB01000016), *C. jukyungi* (TAAB01000018), *A. mori* (AB013087) and *D. citri* (AB038366) from the family Psyllidae, as well as two species from Aphalaridae, *C. fiscella* (KY428658) and *C. maniformis* (KY428659), were included in the analysis. As an out group, we used *Pseudomonas entomophila* (MK214821). The sequences of the endosymbionts from *C. burckhardti C. myrthi*, *C. jukyungi* and *A. mori* were included in the dataset as they are the closest known species to the ASVs of *C. pyri*, *C. pyricola* and *C. pyrisuga* based on a BLAST search. The maximum likelihood (ML) analysis was performed using IQ‐TREE (Trifinopoulos et al., 2016). Nodal support was evaluated using a standard nonparametric bootstrap (BS) with 1000 replicates. Clades recovered with BS > 70% were considered significantly supported. The ML tree was visualized with iTOL (Letunic & Bork, 2021).

## 3. RESULTS

### 3.1. 16S rRNA gene amplicon sequencing

The microbiomes of 151 *Cacopsylla* individuals (*Cacopsylla pyri*, *C. pyricola* and *C. pyrisuga*), as well as three egg clutches from *C. pyrisuga*, were sequenced on the Illumina MiSeq platform. After raw data processing, amplicon sequencing yielded 2352–59,068 high‐quality reads per sample (mean = 24,080), which were clustered into 4–72 amplicon sequence variants (ASVs) per sample (mean = 15.88) (Table S1). Rarefaction curves reached a plateau, indicating that our sequencing effort was sufficient to capture the bacterial diversity of these communities (Figure S3).

After an initial analysis, five immature specimens of *C. pyri* showed very different microbiomes from all other specimens, which led us to test for parasitoid DNA. We detected parasitoid DNA in two out of the five immature individuals of *C. pyri* collected in winter from the site CZ1 (Table S1, highlighted in yellow; Figure S4). Since the microbiomes of the other three immature individuals from the same collection date and site were similar to the microbiomes of the two confirmed parasitized individuals, all five immature specimens of *C. pyri* were discarded from the analyses. No parasitoid DNA was detected in adult specimens of *C. pyri*.

### 3.2. Pear psyllid species harbour different endosymbionts taxa

**FIGURE 2:**
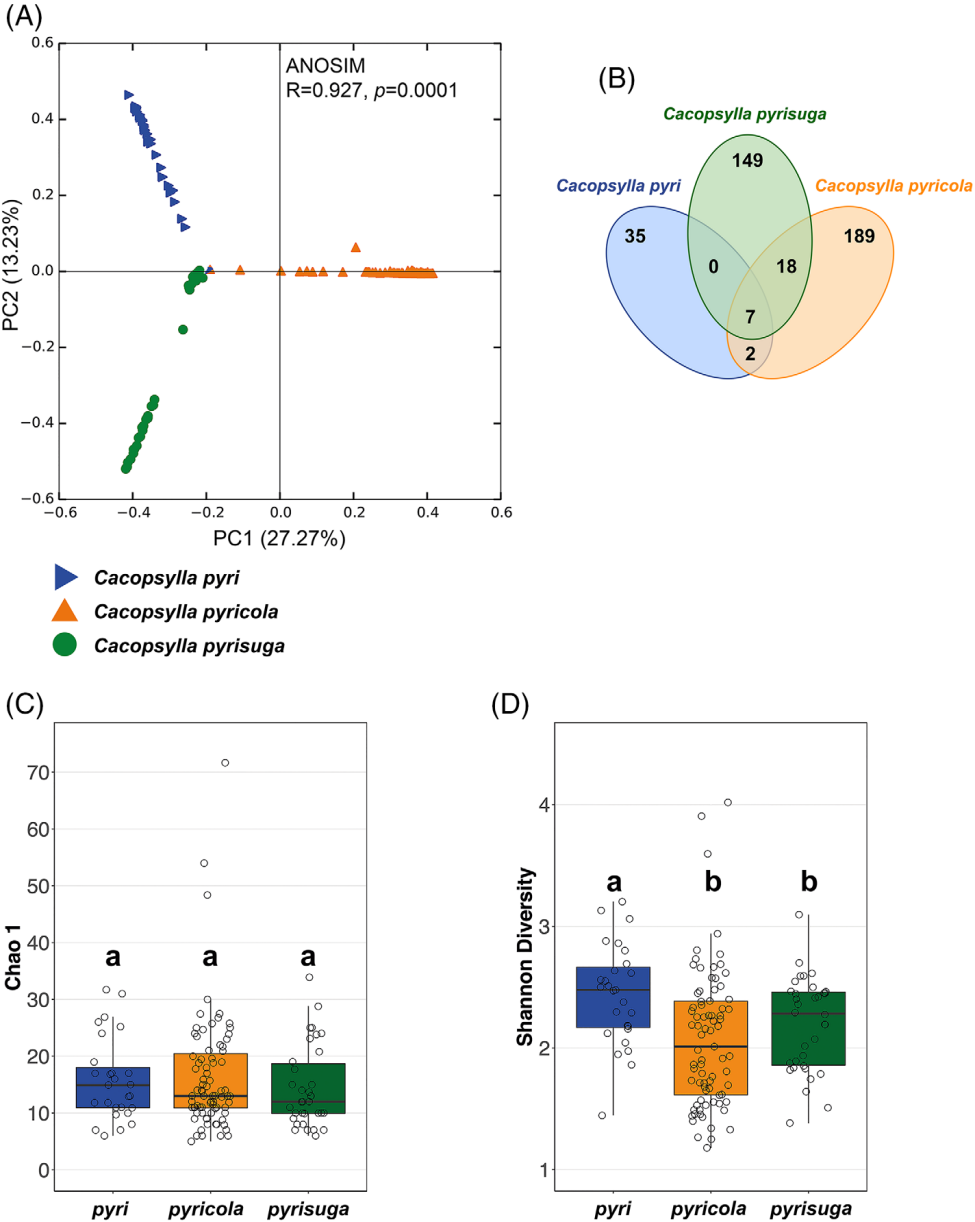
(**A**) Principal coordinates analysis based on bray–Curtis dissimilarity metrics showing differences in microbiome composition between the three *Cacopsylla* species. (**B**) Venn diagram showing the shared and specific bacterial ASVs between adult specimens of all three species co‐occurring in the same locality (CZ2). (**C**) Comparison of bacterial species richness (Chao 1) and (**D**) diversity (Shannon index) between individuals of the three *Cacopsylla* species.

PCoA based on Bray–Curtis distances discriminated the three *Cacopsylla* species from each other, showing that the three pear psyllid species harbour different microbiomes (Figure 2A). Differences in the bacterial species composition among the three *Cacopsylla* species were significant based on ANOSIM (R = 0.927, *p* = 0.0001) and explained 40.50% of the variation between the bacterial communities. When comparing the microbiomes of the three *Cacopsylla* species from a locality where all species co‐occurred (CZ2), only 7 out of 1046 ASVs were found in all three *Cacopsylla* species (Figure 2B). The species which shared the most ASVs were *Cacopsylla pyricola* and *C. pyrisuga* (18), whereas *C. pyri* and *C. pyricola* shared only 2 ASVs, and *C. pyri* and *C. pyrisuga* had no ASVs in common. Hence, even when co‐occurring in the same locality, the three species maintain distinct bacterial communities. Despite the clear differences in microbiome composition, we found no differences in bacterial species richness (Chao 1) between the three species (*C. pyri* vs. *C. pyricola*: *t*‐test = −0.056, *p* = 1.000; *C. pyricola* vs. *C. pyrisuga*: *t*‐test = −1.088, *p* = 0.859; and *C. pyri* vs. *C. pyrisuga*: *t*‐test = −1.194, *p* = 0.701) (Figure 2C). However, the bacterial diversity based on the Shannon index was significantly higher in *C. pyri* compared to both *C. pyricola* (*t*‐test = −3.267, *p* = 0.006), and *C. pyrisuga* (*t*‐test = −2.523, *p* = 0.042) (Figure 2D). Specifically, bacterial diversity reached a mean Shannon index of 2.39 (range = 1.41–3.13) in *C. pyri*, compared to 2.02 (range = 1.17–3.88) in *C. pyricola* and 2.14 (range = 1.36–3.00) in *C. pyrisuga* (Table S1).

In this study, *Carsonella* reads represented only 0%–21.8% of the reads per specimen. The primer set used (341F and 805R) was designed to amplify a wide range of bacteria but contained several mismatches with *Carsonella* in the forward primer (Klindworth et al., 2013; Morrow et al., 2017; Nakabachi et al., 2022). This led to an inefficient amplification of *Carsonella*, but favoured the amplification of other bacterial taxa (consistent with Morrow et al., 2017, 2020 and Kwak et al., 2021).

**FIGURE 3:**
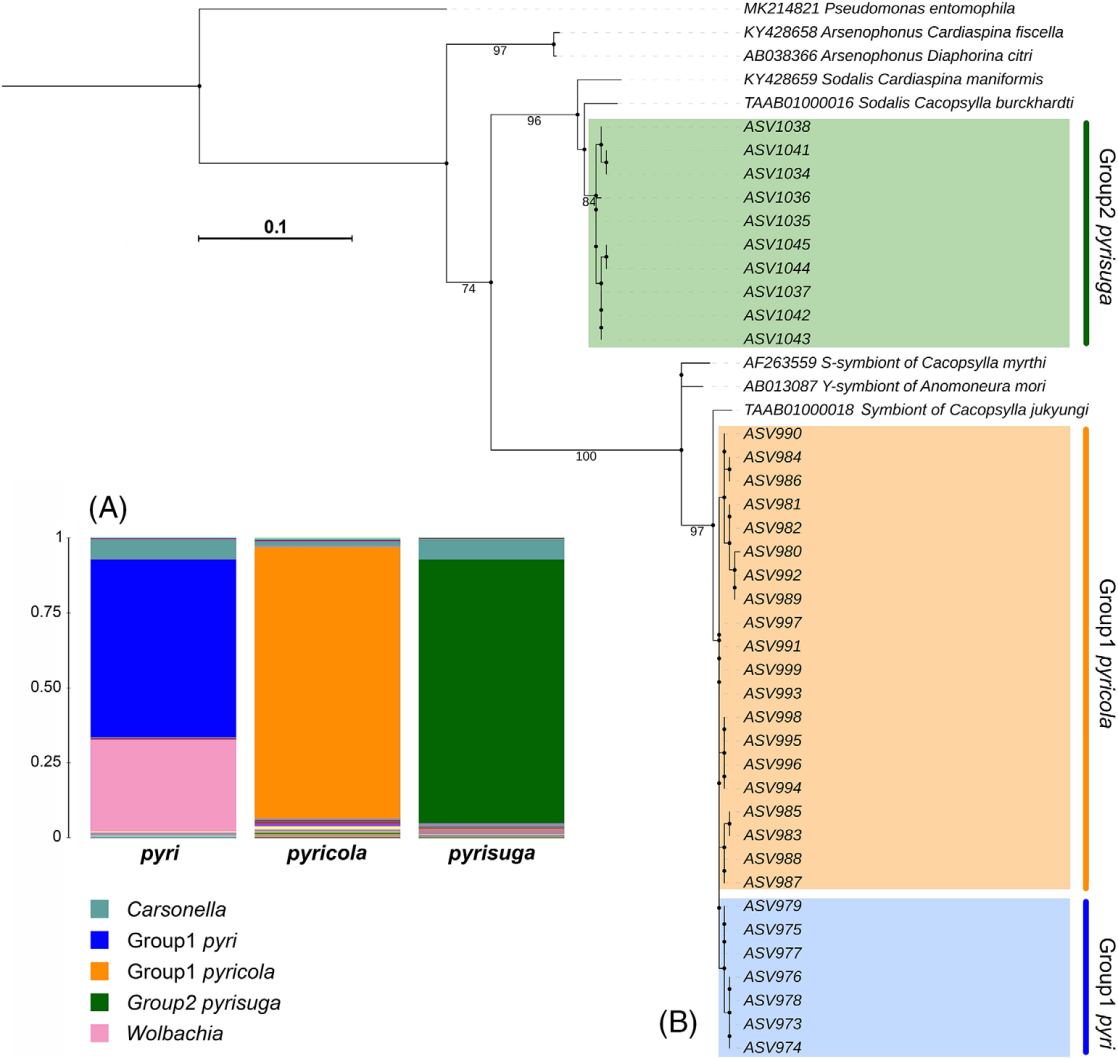
(**A**) Microbiome composition of the three *Cacopsylla* species. The most abundant bacterial genera are represented in the legend. (**B**) Maximum likelihood tree of 16S rRNA gene ASV sequences belonging to the Enterobacteriaceae endosymbionts of pear psyllids. The tree displays the relationships between the endosymbionts Group1 *pyri*, Group1 *pyricola* and Group2 *pyrisuga*, harboured by *Cacopsylla pyri*, *C. pyricola* and *C. pyrisuga*, respectively.

Apart from *Carsonella*, the microbiomes of the studied psyllid species were dominated by very few bacterial taxa. Hence, only 9–20 ASVs per psyllid species corresponded to an abundance >1% of the reads for the given species: 15 ASVs for *C. pyri*, 9 ASVs for *C. pyricola* and 20 ASVs for *C. pyrisuga*. Specifically, all individuals harboured abundant bacteria classified as Enterobacteriaceae (Figure 3A). Both *C. pyri* and *C. pyricola* harboured several ASVs of this endosymbiont, which was given for convenience the following provisional names in our study: ‘Group1 *pyri*’ (58.90% of all reads from *C. pyri*) for ASVs found in *C. pyri* and ‘Group1 *pyricola*’ (89.90% of all reads from *C. pyricola*) for those found in *C. pyricola*. A maximum likelihood phylogenetic analysis of these ASVs and 16S rRNA sequences from other psyllid‐associated endosymbionts showed that Group 1 *pyri* and Group 1 *pyricola* formed a strongly supported clade together with the endosymbiont of *Cacopsylla jukyungi* (BS = 97), which was well‐supported as a sister clade to the endosymbionts of *C. myrthi* and *Anomoneura mori* (BS = 100) (Figure 3B). Likewise, the close relationship with Enterobacteriaceae endosymbionts in *C. myrthi* and *A. mori* had been observed also in other *Cacopsylla* species (Morrow et al., 2020; Schuler et al., 2022), suggesting that these ASVs represent an endosymbiont clade widespread in the subfamily of Psyllinae (Psyllidae). In contrast, the most abundant ASVs assigned to Enterobacteriaceae in *C. pyrisuga* were referred to as “Group2 *pyrisuga*” in our study (87.56% of all reads from *C. pyrisuga*). Group2 *pyrisuga* showed its close relatedness to the *Sodalis* endosymbionts of *Cacopsylla burckhardti* and *Cardiaspina maniformis* (Aphalaridae) (BS = 96) (Figure 3B). Together, Group2 *pyrisuga* and the *Sodalis* endosymbionts of *C. burckhardti* and *C. maniformis* were placed as a sister clade to the one formed by Group1 *pyri*, Group1 *pyricola* and the endosymbionts of *C. jukyungi*, *C. myrthi* and *A. mori* (BS = 74). Last, *Arsenophonus* endosymbionts of *C. fiscella* (Aphalaridae) and *D. citri* (Psyllidae) established a well‐supported monophyletic group (BS = 97) that was placed as a sister to all analysed bacterial taxa. Taken together, our results indicate that the three studied psyllid species each harbour a highly abundant endosymbiont and that *C. pyrisuga* harbours a different taxon compared to the endosymbiont of *C. pyri* and *C. pyricola*.

**FIGURE 4:**
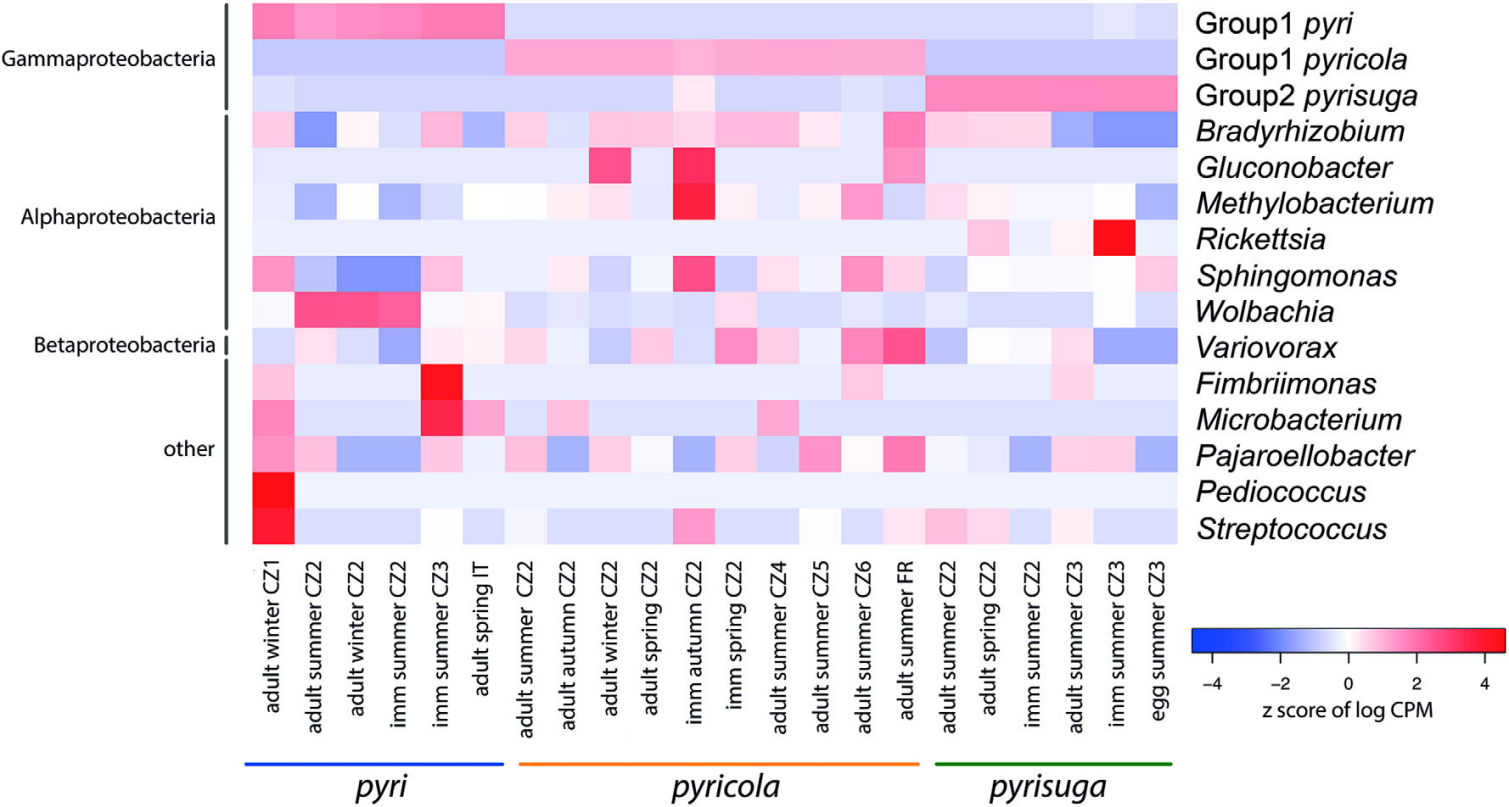
Heatmap showing the abundance (log counts per million) of 15 bacterial genera identified as either indicator species and/or differentially abundant between the three *Cacopsylla* species. Each column represents several psyllid individuals that are grouped together based on their developmental stage, generation (seasonality) and geographic location. Bacterial genera are ordered based on class level designations represented on the left of the heatmap. Psyllid species are indicated by the coloured bar below the heatmap.

To better understand which bacterial taxa were responsible for the differences between the microbiomes of the three *Cacopsylla* species, we determined indicator species (using the R function IndVal) and differentially abundant bacterial taxa (edgeR). The IndVal analysis detected 10 bacterial taxa as potential indicator taxa which were predominantly present in only one psyllid species: six taxa in *C. pyri*, and two taxa each for *C. pyricola* and *C. pyrisuga*. These corresponded to Group1 *pyri*, *Wolbachia*, *Streptococcus*, *Pediococcus*, *Microbacterium* and *Fimbriimonas* for *C. pyri*; Group 1 *pyricola* and *Gluconobacter* for *C. pyricola*; and Group 2 *pyrisuga* and *Rickettsia* for *C. pyrisuga* (Figure 4). Five additional bacterial taxa were identified as differentially abundant between the three psyllid species. Specifically, *Bradyrhizobium* and *Methylobacterium* were more abundant in *C. pyricola* compared to *C. pyri* and *C. pyrisuga*, and *Sphingomonas* and *Variovorax* were more abundant in *C. pyricola* compared only to *C. pyrisuga*. *Pajaroellobacter* was more abundant in *C. pyri* and *C. pyricola* compared to *C. pyrisuga* (Figure 4).

### 3.3. Impact of host biology and geography on psyllid microbiome

**FIGURE 5:**
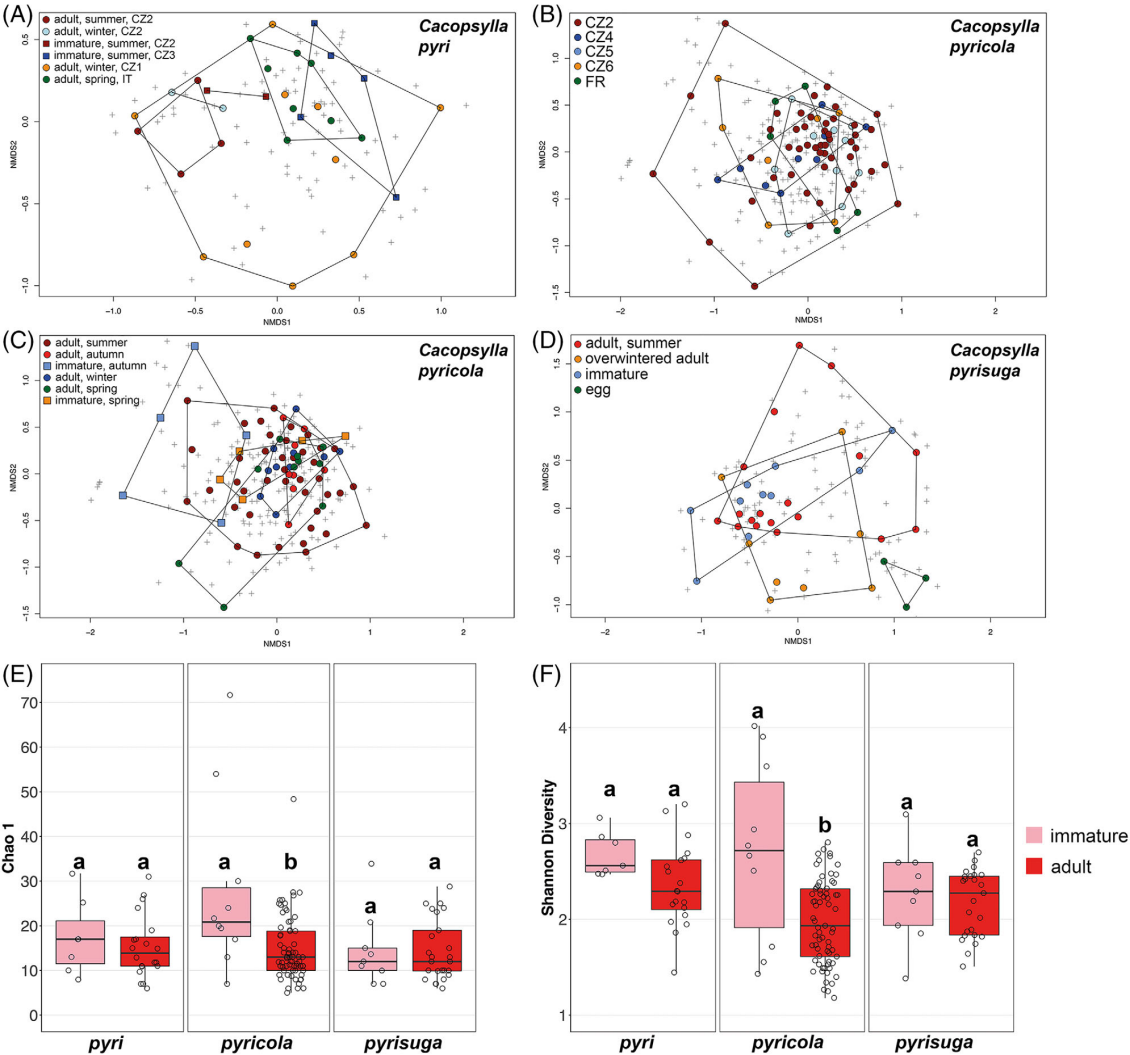
Impact of host life history traits on the bacterial communities of the three *Cacopsylla* species visualized with nonmetric multidimensional scaling. Datapoints are coloured based on the tested life history traits. Group boundaries were drawn using the ordihull function (R package ‘vegan’). (**A**) Microbiome composition of *C. pyri* individuals along host ontogeny, generations and localities. (B) Microbiome composition of *C. pyricola* individuals from different localities. (C) Microbiome composition of *C. pyricola* individuals along host ontogeny and generations. (D) Microbiome composition of *C. pyrisuga* individuals along host ontogeny and seasonality. Comparison of bacterial species richness (E) and diversity (F) between immature and adult individuals of the three *Cacopsylla* species. Different letters indicate significant differences based on *t*‐tests.

**FIGURE 6:**
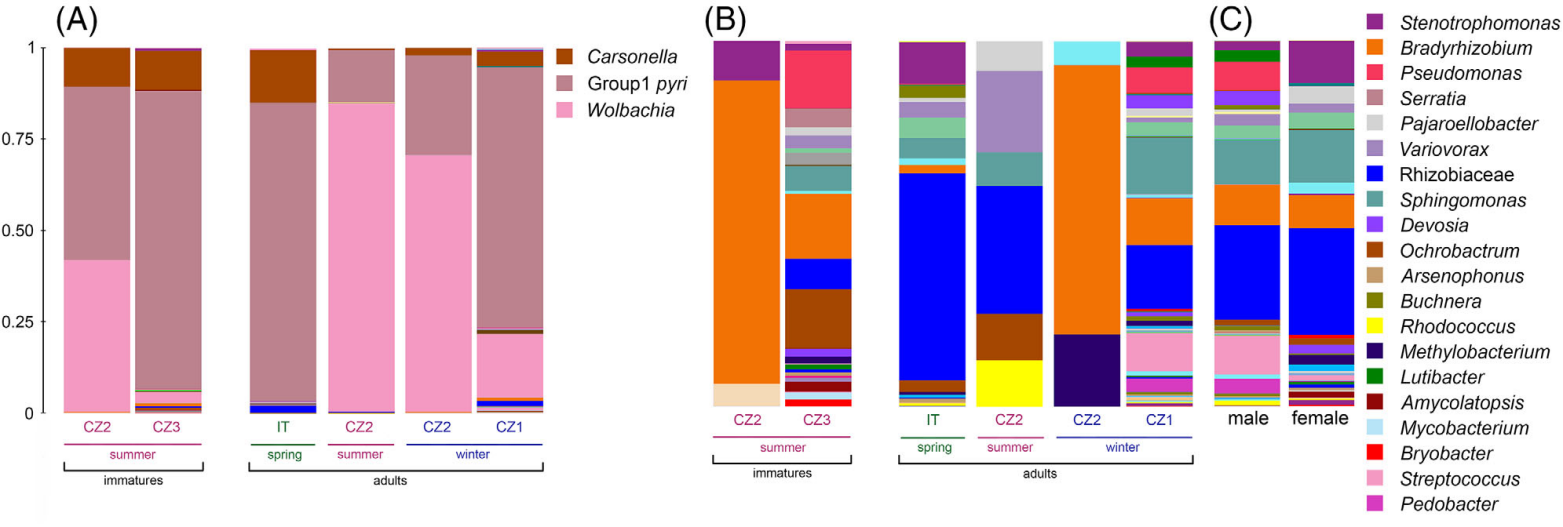
Microbiome composition of *Cacopsylla pyri* depending on developmental stages, generations and localities. The most abundant bacterial genera are represented in the legend. (A) Microbiome composition including the most abundant taxa *Carsonella*, Group1 *pyri* and *Wolbachia* and (B) after removing ASVs of *Carsonella*, Group1 *pyri* and *Wolbachia*. (C) Comparison of microbiome composition between males and females after removing ASVs belonging to *Carsonella*, Group1 *pyri* and *Wolbachia*. Detailed information on all samples is provided in Table S1.

**FIGURE 7:**
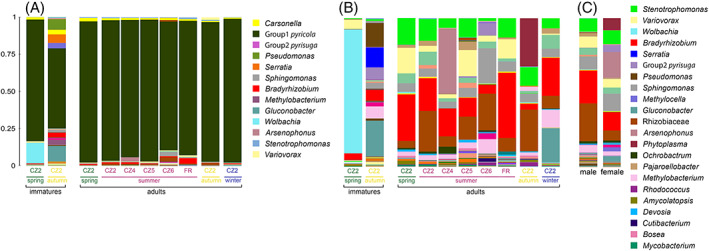
Microbiome composition of *Cacopsylla pyricola* depending on developmental stages, generations and localities. The most abundant bacterial genera are represented in the legend. (A) Microbiome composition including the most abundant taxa *Carsonella* and Group 1 *pyricola* and (B) after removing ASVs of *Carsonella* and Group 1 *pyricola*. (C) Comparison of microbiome composition between males and females after removing ASVs belonging to *Carsonella* and Group1 *pyricola*. Detailed information on all samples is provided in Table S1.

**FIGURE 8:**
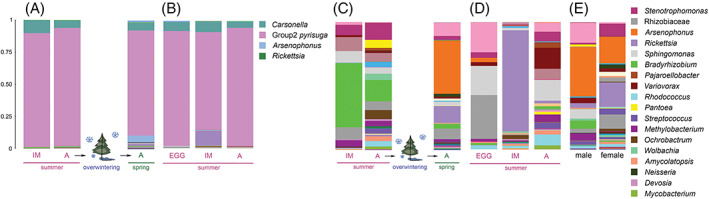
Microbiome composition of *Cacopsylla pyrisuga* depending on developmental stages, seasonality and localities. The most abundant bacterial genera are represented in the legend. (A) Microbiome composition across developmental stages of *C. pyrisuga* from a single locality (CZ2) including *Carsonella* and Group 2 *pyrisuga* and (C) after removing ASVs of *Carsonella* and Group2 *pyrisuga*. (B) Microbiome composition across developmental stages of *C. pyrisuga* from a single locality (CZ3) including *Carsonella* and Group 2 *pyrisuga* and (D) after removing ASVs of *Carsonella* and Group 2 *pyrisuga*. (E) Comparison of microbiome composition between males and females after removing ASVs of *Carsonella* and Group 2 *pyrisuga*. Detailed information on all samples is provided in Table S1.

The effect of different developmental stages, generations for polyvoltine species, seasonality for univoltine species, sex and different geographical localities on microbiome structure were tested for each of the three *Cacopsylla* species. Correlations between these factors and differences in microbiome composition (beta diversity) were evaluated using EnvFit (Table 2) applied to NMDS analyses based on Bray–Curtis distances (Figures 5A–D). The species richness and diversity indices (Chao 1 and Shannon, respectively) were used to detect differences in alpha diversity depending on the above‐mentioned factors (Table S3; Figures 5E–F). All analyses were performed for each psyllid species separately due to the substantial differences in their biology and sampling sites. There were no differences in microbiome composition between males and females in any of the studied psyllid species (Table 2; Figures 6C, 7C and 8E).

**TABLE 2:** EnvFit results for each *Cacopsylla* species applied to nonmetric multidimensional scaling (NMDS) based on Bray–Curtis distances, with the following categories examined: Developmental stage, generation (seasonality), locality and sex. Significant *p*‐values are in bold

Psyllid species	Developmental stage	Generation (seasonality)	Developmental stage + generation	Locality	Sex
*Cacopsylla pyri*	*r* ^2^ = 0.053, *p* = 0.191, stress = 0.165	*r* ^2^ = 0.089, *p* = 0.263, stress = 0.165	*r* ^2^ = 0.243, ***p* = 0.004**, stress = 0.165	*r* ^2^ = 0.330, ***p* = 0.001**, stress = 0.165	*r* ^2^ = 0.054, *p* = 0.319, stress = 0.140
*Cacopsylla pyricola*	*r* ^2^ = 0.089, ***p* = 0.002**, stress = 0.206	*r* ^2^ = 0.040, *p* = 0.453, stress = 0.206	*r* ^2^ = 0.164, ***p* = 0.005**, stress = 0.206	*r* ^2^ = 0.032, *p* = 0.775, stress = 0.206	*r* ^2^ = 0.011, *p* = 0.478, stress = 0.208
*Cacopsylla pyrisuga*	*r* ^2^ = 0.036, *p* = 0.614, stress = 0.144	*r* ^2^ = 0.073, *p* = 0.065, stress = 0.144		*r* ^2^ = 0.006, *p* = 0.833, stress = 0.144	*r* ^2^ = 0.075, *p* = 0.143, stress = 0.124

#### 3.3.1. The microbiome of *C. pyri* varied across development, seasonal generations and geography

We found significant differences in microbiome composition between different developmental stages, generations and localities for *C. pyri* (Table 2; Figure 5A). Notably, the interaction between host developmental stages and generations was significant (EnvFit: *r* ^2^ = 0.243, *p* = 0.004), while both factors were not significant when tested independently (Table 2). The effect of geographical localities on the microbiome was tested for four populations of *C. pyri* collected in two countries (Czech Republic, Italy) (Tables 1 and S1). The locality was the most significant factor shaping microbiome composition, even when the most distant population collected in Italy (IT) was excluded from the analysis to evaluate only the differences between the Czech populations (EnvFit: *r* ^2^ = 0.330, *p* = 0.001). A major difference between the different localities was that individuals from the Czech populations CZ1, CZ2 and CZ3 harboured *Wolbachia* at varying relative abundances (20.19%, 65.42% and 3.02%, respectively), whereas only traces of *Wolbachia* (0.10%) were found in the IT population (Figures 6A and S4).

In total, the microbiome of *C. pyri* contained 20 ASVs assigned to *Wolbachia* (Table S2). Eight were present in all specimens (P1–P8) collected at the site CZ2 (Figure S4A), which represented different developmental stages and generations (Table S1). The relative abundance of *Wolbachia* varied with host ontogeny when comparing specimens collected from the same locality (CZ2) (Figure 6A). *Wolbachia* was more abundant in adults from the summer (84.43%) and overwintering generation (70.29%) compared to summer‐form immatures (41.54%). At the site CZ1, *Wolbachia* was found in a single male individual (P20) which exhibited a very high *Wolbachia* abundance (91.47% of all reads from this specimen) represented by 3 ASVs identical to the ones harboured by the individuals from CZ2 (Table S2; Figure S4A). In contrast, *Wolbachia* from the site CZ3 comprised a single ASV different from those found at the sites CZ1 and CZ2 (Table S2). To obtain a better picture of the more abundant bacterial taxa apart from the predominating endosymbionts Group1 *pyri*, *Carsonella* and *Wolbachia*, their ASVs were removed from the dataset (Figures 6B and S4B). This revealed several abundant taxa assigned either to the genus *Bradyrhizobium* or to the family Rhizobiaceae. Both ASV groups were present in most individuals but fluctuated in relative abundance between different generations and localities (Figures 6B and S4B).

#### 3.3.2. The microbiome of *Cacopsylla pyricola* varied across development and seasonal generations but not geography

In *Cacopsylla pyricola*, geography had no significant effect on its bacterial community structure despite being tested on five populations collected in two countries (Czech Republic, France) (Tables 2 and S4; Figure 5B). However, as for *C. pyri*, in *C. pyricola* the interaction between host developmental stages and generations was correlated with changes in microbiome composition (EnvFit: *r* ^2^ = 0.164, *p* = 0.005) (Table 2; Figure 5C). Notably, host developmental stages had a significant impact on microbiome composition on its own (EnvFit: *r* ^2^ = 0.089, *p* = 0.002) (Table 2; Figure 5C). In addition, bacterial species richness and diversity were higher in immatures than in adults (Chao 1: *t*‐test = −3.966, *p* = 0.001; Shannon: *t*‐test = −4.191, *p* = 0.002) (Figures 5E–F). In contrast, the effect of different generations was significant only in combination with host developmental stages (Table 2; Figure 5C), suggesting that changes in microbiome composition between different generations occurred in only one developmental stage, either immatures or adults, but not in both. Considering the NMDS pattern, this seems more likely for immatures, since immatures collected in spring and autumn do not cluster together (Figure 5C). This suggests that differences in microbiome composition were most strongly correlated with host development in this species. This pattern could be explained by the high abundance of *Wolbachia* only in spring‐form immatures (83.19% of reads after removing the ASVs of the predominant endosymbionts), whereas no *Wolbachia* was found in autumn‐form immatures from the same site, nor in adults of *C. pyricola* (Figures 7A–B). Contrary to spring‐forms (Figures 7A–B), autumn‐form immatures harboured several other abundant bacterial taxa, such as *Gluconobacter* (24.01%), *Pseudomonas* (15.58%) and *Serratia* (13.46%). Bacterial species richness and diversity were also significantly higher in autumn‐form compared to spring‐form immatures (mean Chao 1 = 35.998, mean Shannon = 3.130 for autumn‐form immatures; and mean Chao 1 = 14.975, mean Shannon = 2.139 for spring‐form immatures) (Table S1). In contrast to immatures, *Gluconobacter*, *Pseudomonas* and *Serratia* were not detected in autumn‐form adults which, however, carried higher relative abundances of unidentified Rhizobiaceae (27.86%) and *Phytoplasma* (32.46%) (Figure 7B). The latter was found in a single female where *Phytoplasma* reached an abundance of 93% of all reads (Figure S5B). Surprisingly, adults collected in winter showed a similarly high relative abundance of *Gluconobacter* as immatures (22.96%), but no *Pseudomonas* and *Serratia* (Figure 7B). Taken together, these results suggest that most changes in microbiome composition in *C. pyricola* occur in immatures, whose microbiomes differed between seasons and were different from the microbiomes of adults from the same season.

#### *3.3.3.* Developmental stage, seasonality and geography had no effect on microbiome of *Cacopsylla pyrisuga*

In *C. pyrisuga*, neither host developmental stages, seasonality nor geography were significantly correlated with changes in bacterial species richness, diversity and microbiome composition (Tables 2 and S4, Figures 5E,F), suggesting that the bacterial community of *C. pyrisuga* remains relatively stable during the psyllid life cycle and between populations from different localities. Nonetheless, there may be a trend towards a different microbiome composition in eggs compared to other developmental stages, as seen by the clustering of the egg microbiome when visualized employing the NMDS ordination (Figure 5D). Apart from Group2 *pyrisuga* and *Carsonella* (Figures 8A–B and S6A), several bacterial genera reached relatively high relative abundances in this species, namely *Bradyrhizobium*, *Arsenophonus*, *Rickettsia*, *Sphingomonas* and unclassified Rhizobiaceae (Figures 8C,D and S6B). The latter two taxa reached high proportions (22.86% and 34.40%, respectively) in the three egg samples from the site CZ3 (Figure 8D). Compared to the eggs, a high relative abundance of *Rickettsia* (79.35%), represented by three ASVs, was found in immatures from the same locality (Table S2). During the host ontogeny from immature to adult, *Rickettsia* declined drastically, while *Variovorax* (16.55%) and *Sphingomonas* (16.32%) increased in relative abundance (Figure 8D). At the immature stage, these genera were either absent or present at low relative abundance. Minor compositional differences were detected between immatures and summer‐form adults from site CZ2 (Figure 8C). Interestingly, only overwintered adults collected on pears from the same site in spring harboured *Arsenophonus* (41.74%), followed by *Rickettsia* (13.32%) (Figure 8C). The latter occurred in a single female, reaching 93.15% relative abundance in this particular specimen (Figure S6B).

## 4. DISCUSSION

Our study characterized and compared the microbiomes of the three psyllid species *Cacopsylla pyri*, *C. pyricola* and *C. pyrisuga* associated with pear trees in Central Europe. The microbiomes of the three species were clearly distinct from one another, which led us to investigate whether differences in host life cycle, developmental stages, sex and geography could lead to changes in the bacterial community structure of the insect host (Gonella et al., 2019; McLean et al., 2019). We also examined the phylogenetic relationships of the prevailing endosymbionts within the studied psyllid species. Overall, our metabarcoding approach captured three different types of psyllid‐associated bacteria: (i) Bacteriome‐associated primary endosymbionts which provide the host with essential nutrients, (ii) facultative intracellular bacteria which can be localized in various host tissues and may have beneficial, neutral or detrimental effects on the host, and (iii) extracellular bacteria, notably within the insect gut.

### 4.1. Impact of host biology, ontogeny and geography on pear psyllid microbiomes

We found that the microbiomes of *C. pyri* and *C. pyricola* were more variable compared to the microbiome of *C. pyrisuga*. This was most pronounced in *C. pyri*, whose microbiome composition varied between different localities but also along host ontogeny and between different seasonal generations. In *C. pyricola*, the microbiome underwent major changes during development towards a lower species richness and diversity at the adult stage. Similarly, the bacterial diversity in the Asian Citrus psyllid *D. citri* showed a declining tendency towards the adult stage (Meng et al., 2019), and the bacterial richness in the tobacco whitefly *Bemisia tabaci* was found to be highest in first instar nymphs (Indiragandhi, 2010). We can only hypothesize about the mechanisms underlying the shifts in microbiome structure throughout the ontogenetic development of psyllids. For example, following recent studies on feeding behaviour (George et al., 2018; Görg & Gross, 2021), immatures of psyllids spend more time ingesting phloem than adults. This longer feeding time on nutrient‐rich phloem might explain the higher bacterial richness and diversity found in immatures of *C. pyricola*. Another plausible explanation of varying bacterial richness and diversity along host life cycle might be differences in host physiology (feeding behaviour, immune response, maturation of the reproductive organs) between different life stages (Arp et al., 2016; Inoue et al., 2009; Kandasamy et al., 2022; Nishide et al., 2019).

*Cacopsylla pyri* and *C. pyricola* are two species with very similar biology, but different from *C. pyrisuga*: both species produce several generations per year and overwinter predominantly on pear trees. Interestingly, the studies by Horton et al. (1992, 1994) on the biology of *C. pyricola* from North America, where this species was introduced from Europe, demonstrated that some individuals overwinter on conifers or in apple orchards, while other individuals from the same population stay in pear orchards throughout an entire year. In contrast to *C. pyri* and *C. pyricola*, *C. pyrisuga* is a univoltine species that has an obligate overwintering association with conifers (Hodkinson, 2009; Lauterer, 1999). This suggests that the presence of only one generation in *C. pyrisuga* or its obligate overwintering on shelter plants may contribute to the relative stability of microbiome of *C. pyrisuga*.

In contrast to *C. pyri* and *C. pyricola*, in *C. pyrisuga* we found no changes in the bacterial community structure depending on the tested factors (developmental stages, seasonality and geography). *C. pyrisuga* had not been considered a competent vector of ‘*Ca*. Phytoplasma mali’ (Jarausch et al., 2019; Jarausch & Jarausch, 2010), until Riedle‐Bauer et al. (2022) recently demonstrated experimentally that remigrants of *C. pyrisuga* are able to transmit ‘*Ca*. Phytoplasma mali’ to pear trees in Austria. Although naturally infected individuals of *C. pyrisuga* were previously found in the Czech Republic, their ability to transmit phytoplasma has not yet been proven there (Kučerová et al., 2007). In a similar manner, the apple psyllid *Cacopsylla melanoneura* was shown to transmit ‘*Ca*. Phytoplasma mali’ in Northern Italy but not in Germany (Mayer et al., 2009; Tedeschi et al., 2012). Thus, the capability to transmit phytoplasma might vary across regions. Therefore, transmission trials are needed to validate a putative vector competence of *C. pyrisuga* across its distributional range. Future research should also test if the stability in the microbiome structure of *C. pyrisuga* from Czech populations or the presence of *Arsenophonus* or a *Sodalis*‐like potential co‐primary endosymbiont in the microbiome could contribute to its vector incompetence/competence in this area.

### 4.2. Major facultative symbionts of the studied *Cacopsylla* species

In our study, *Wolbachia* was found at a high relative abundance only in *Cacopsylla pyri*, particularly at the adult stage. This is consistent with results from other studies on psyllid microbiomes, demonstrating that *Wolbachia* abundance tends to increase towards the adult stage (Meng et al., 2019; Ramsey et al., 2017). This is thought to be associated with high nutritional demands and organogenesis during psyllid development (Ramsey et al., 2017). *Wolbachia* is a common bacterium, infecting 40%–52% of terrestrial arthropods, and is mostly maternally inherited (Kaur et al., 2021; Weinert et al., 2015). Although *Wolbachia* maintains nutritional mutualistic associations with some hemipteran hosts (Hosokawa et al., 2010), most studies on insects demonstrated *Wolbachia*‐induced reproductive manipulations (Engelstädter & Hurst, 2009; Schuler et al., 2016), and/or protection against pathogens (Hedges et al., 2008; Teixeira et al., 2008). In psyllids, the role of *Wolbachia* has been explored only in several pest species, despite *Wolbachia*'s ubiquitous distribution in this group (Fromont et al., 2016, 2017; Fu et al., 2020; Morrow et al., 2017, 2020; Nakabachi et al., 2022; Overholt et al., 2015). Particularly, previous studies on *D. citri* demonstrated a link between *Wolbachia* infection and ‘*Ca*. Liberibacter asiaticus’ titers, indicating that *Wolbachia* may both mitigate and impede the transmission of the pathogen in *D. citri* (Fagen et al., 2012; Hosseinzadeh et al., 2019; Kruse et al., 2017; Song et al., 2019). Likewise, *Wolbachia* strains that might induce cytoplasmatic incompatibility were detected in the potato psyllid *B. cockerelli* (Cooper, Swisher, et al., 2015; Fu et al., 2020; Hail et al., 2012; Nachappa et al., 2011). Considering the major biological roles that *Wolbachia* plays in other insect taxa (Bourtzis et al., 2014; Chrostek et al., 2013; Landmann, 2019; Nikoh et al., 2014; Weinert et al., 2015), its impact on biology and vector competence of *C. pyri* is of great interest.

*Arsenophonus* was observed in this study in high relative abundance only in overwintered adults (remigrants) of *C. pyrisuga*, although it was also detected at low abundance in several individuals of *C. pyri* and *C. pyricola*. Like other univoltine species that overwinter on conifers, that is, *Cacopsylla pruni* and *C. picta* (Barthel et al., 2020; Gallinger & Gross, 2018), *C. pyrisuga* can probably feed on conifer sap. The additional food source broadens the potential bacterial influx. In theory, both the conifer sap and the pear tree phloem (Gallinger & Gross, 2018) might mediate horizontal transmission of different bacteria among various insects associated with these plants. The genus *Arsenophonus* is known for its diversity and frequent horizontal transmissions between distantly related insect species (Jousselin et al., 2013; Mouton et al., 2012; Russell et al., 2003). Its broad range of symbiotic relationships encompasses both beneficial and manipulative associations (Ferree et al., 2008; Gherna et al., 1991; Hansen et al., 2007; McCutcheon et al., 2019; Nováková et al., 2009; Šochová et al., 2017). While *Arsenophonus* was previously detected in various psyllid species (Hall et al., 2016; Morrow et al., 2017; Nakabachi et al., 2022; Schuler et al., 2022; Sloan & Moran, 2012; Subandiyah et al., 2000; Thao et al., 2000), potentially as an obligate or at least beneficial symbiont (Hall et al., 2016; Hansen et al., 2007; Morrow et al., 2017; Subandiyah et al., 2000), its role in *C. pyrisuga*, particularly in remigrants that could transmit phytoplasma, remains to be elucidated.

To date, only a few studies have reported *Rickettsia* in psyllids (Cooper et al., 2021; Morrow et al., 2020; Nakabachi et al., 2022; Pilgrim et al., 2021; Schuler et al., 2022). We showed both immatures and overwintered adults of *C. pyrisuga* to harbour *Rickettsia*. Previously, mostly known as vector‐borne pathogens of vertebrates, *Rickettsia* are intracellular bacteria associated with a broad range of arthropods including phloem‐feeding hemipterans (Pilgrim et al., 2021; Wang et al., 2020; Weinert et al., 2015). Some hemipteran strains of *Rickettsia* have profound effects on insect biology and vector–pathogen interactions (Kliot et al., 2014; Morrow et al., 2020; Pilgrim et al., 2021; Wang et al., 2020). For instance, it was demonstrated that *Rickettsia* manipulates the transmission efficiency of the Tomato yellow leaf curl virus by the whitefly *Bemisia tabaci* (Kliot et al., 2014). This highlights the importance of future studies on potential effects of *Rickettsia* and *Arsenophonus* associates pathogen transmission abilities of *C. pyrisuga*.

Apart from these facultative intracellular symbionts, we also detected numerous other bacterial taxa, many of which likely represented extracellular bacteria from the insect gut. Despite being captured only at low abundance compared to the predominating intracellular symbionts, some of these were identified as species‐specific (*Fimbriimonas*, *Gluconobacter*, *Microbacterium*, *Pediococcus* and *Streptococcus*) or differentially abundant taxa (*Bradyrhizobium*, *Methylobacterium*, *Pajaroellobacter*, *Sphingomonas* and *Variovorax*), thereby contributing to the differences between the microbiomes of the three species. However, to date nothing is known regarding the potential roles of these bacteria in psyllids.

### 4.3. Potential co‐primary endosymbionts of pear psyllids and their phylogenetic relationships

Like other groups of sap‐feeding insects, psyllids maintain an obligatory association with the primary endosymbiont ‘*Candidatus* Carsonella ruddii’, which synthesizes essential amino acids and thus compensates for their deficiency in the phloem sap (Nakabachi et al., 2006; Sloan & Moran, 2012; Tamames et al., 2007). *Carsonella* resides in specialized host cells, bacteriocytes, which constitute a tissular structure called a bacteriome. It appears to be universally present in psyllids and represents a case of strict host–symbiont co‐speciation, as a result of vertical transmission and long‐term evolutionary co‐divergence between the endosymbiont and its insect host (Fromont et al., 2016; Hall et al., 2016; Thao et al., 2000). In addition to the obligate primary (P‐) endosymbiont *Carsonella*, numerous other secondary endosymbionts were detected in various psyllids. Depending on the psyllid species, these belong to various bacterial groups, notably Betaproteobacteria (*Profftella*) and Enterobacteriaceae (*Arsenophonus*, *Sodalis*, unclassified Enterobacteriaceae) (Fromont et al., 2016, 2017; Morrow et al., 2017, 2020;Nakabachi et al., 2013 ; Nakabachi, Malenovský, et al., 2020 ; Nakabachi, Piel, et al., 2020). Interestingly, some of these endosymbionts were shown to occur in the syncytium of the bacteriome, that is, they co‐inhabit the bacteriome together with *Carsonella* and can therefore be vertically transmitted (Fukatsu & Nikoh, 1998; Nakabachi et al., 2013; Sloan & Moran, 2012; Subandiyah et al., 2000). Hence, these secondary endosymbionts may in fact act as co‐primary (CP‐) nutritional and/or defensive endosymbionts with reduced genomes due to their obligate host‐associated lifestyle (Nakabachi et al., 2013; Nakabachi, Piel, et al., 2020; Sloan & Moran, 2012). In contrast to *Carsonella*, other endosymbionts such as *Arsenophonus* and *Sodalis* exhibit patterns of past horizontal transmission, suggesting more recent independent acquisitions and/or occasional host shifts (Fromont et al., 2016; Hall et al., 2016).

In our study, we detected Enterobacteriaceae in all analysed specimens of the three *Cacopsylla* species. These endosymbionts were recovered at a very high relative abundance and were identified as unclassified Enterobacteriaceae that are not yet formally described. Similarly, Morrow et al. (2017, 2020), Kwak et al. (2021), Nakabachi et al. (2022) and Schuler et al., 2022 found endosymbionts referred to as unclassified Enterobacteriaceae in several *Cacopsylla* species. Altogether, unclassified Enterobacteriaceae were detected in six out of seven currently recognized psyllid families (cf. Burckhardt et al., 2021), namely Aphalaridae, Calophyidae, Carsidaridae, Liviidae, Psyllidae and Triozidae (Fromont et al., 2017; Kwak et al., 2021; Morrow et al., 2017, 2020; Nakabachi et al., 2022; Overholt et al., 2015). Our study showed that *C. pyri* and *C. pyricola* share similar Enterobacteriaceae endosymbionts, closely related to the endosymbionts of *C. jukyungi* (the latter species is also associated with pear trees but distributed in eastern Asia; Cho et al., 2017, 2020), *C. myrthi* and *A. mori*, while the endosymbionts of *C. pyrisuga* and *C. burckhardti* are more closely related to the genus *Sodalis*. Nakabachi et al. (2022) identified the endosymbiont of *C. burckhardti* as *Sodalis* given that its ASVs showed 94.6%–97.9% identity to previously identified *Sodalis* from various insect groups. Given that *C. pyrisuga* and *C. burckhardti* are two closely related species, both associated with pear trees (Cho et al., 2017, 2020), it is interesting to note that their endosymbionts also seem to be closely related and might represent a new *Sodalis*‐like lineage. However, because our results on the phylogenetic relationships of Enterobacteriaceae endosymbionts of the studied *Cacopsylla* species were based on short fragments of the 16S rRNA gene, this should be interpreted with caution. Considering the occurrence of Enterobacteriaceae endosymbionts at high abundance in all analysed psyllid specimens as well as in other psyllid taxa (e.g., Kwak et al., 2021; Morrow et al., 2017, 2020; Nakabachi et al., 2022; Overholt et al., 2015; Schuler et al., 2022), their presence throughout the entire psyllid life cycle and relatedness to the endosymbionts of *C. myrthi* and *A. mori*, which are known to be localized in the bacteriome together with *Carsonella*, it is likely that the Enterobacteriaceae endosymbionts recovered from *C. pyri*, *C. pyricola* and *C. pyrisuga* are vertically transmitted, obligate co‐primary (CP‐) endosymbionts. However, to validate our hypothesis, the metabolic potential and tissular localization of the Enterobacteriaceae endosymbionts still need to be elucidated.

Based on the phylogenomic study of Psylloidea by Percy et al. (2018), all *Cacopsylla* species associated with Rosaceae, including *C. pyri*, *C. pyricola* and *C. pyrisuga*, belong to one clade. In turn, *C. myrthi* associated with the plant family Rhamnaceae is a member of a different *Cacopsylla* species group, whereas *A. mori* belongs to a more distantly related genus of Psyllidae. Considering this pattern of phylogenetic relationships within Psyllidae (Cho et al., 2020; Percy et al., 2018), the phylogeny of the Enterobacteriaceae endosymbionts does not fully match the phylogeny of their hosts. This is in line with previously observed incongruences between phylogenies of psyllids and their secondary endosymbionts (CP‐endosymbionts in the present study) (Hall et al., 2016; Thao et al., 2000; Thao & Baumann, 2004) and supports the hypothesis of past horizontal transfers of the secondary endosymbionts and their host shifts throughout the evolutionary history of psyllids.

According to our results, the most striking distinction between the studied pear psyllids lies in the presence of their dominant Enterobacteriaceae endosymbionts. As shown in several studies on insect microbiomes, various endosymbionts can play different functional roles in their hosts (Gottlieb et al., 2010; Hansen et al., 2007; Nakabachi et al., 2020b; Nikoh et al., 2014). For instance, a defensive role was demonstrated for *Profftella*, which co‐occurs with *Carsonella* in the bacteriome of *Diaphorina* spp. (Nakabachi et al., 2013; Nakabachi, Malenovský, et al., 2020; Nakabachi, Piel, et al., 2020). Future investigations of the biological role(s) of CP‐endosymbionts of pear psyllids using genomics would be necessary to better understand their functional and metabolic potential. This will also contribute to a better understanding of the relationships between CP‐endosymbionts and their psyllid hosts across the psyllid tree of life.

## AUTHOR CONTRIBUTIONS

Liliya Štarhová Serbina and Hannes Schuler conceived the project; Liliya Štarhová Serbina, Domagoj Gajski and Igor Malenovský collected and identified the psyllid specimens; Liliya Štarhová Serbina, Ludek Zurek and Eva Nováková organized the data; Liliya Štarhová Serbina, Domagoj Gajski and Barbora Pafčo conducted molecular analyses; Jessica Dittmer supervised bioinformatics and statistical analyses; Liliya Štarhová Serbina and Jessica Dittmer performed data analysis; Liliya Štarhová Serbina prepared the first draft and wrote the manuscript together with Jessica Dittmer. All authors provided feedback that helped to improve the manuscript.

## Supporting information


**Figure S1** Habitus of adult *Cacopsylla pyri* – female (A), *C. pyricola* – female, summer morph (B) and *C. pyrisuga* – male, freshly emerged, early summer individual (C), and immature *C. pyricola* (D) and *C. pyrisuga* (E). The photos from the living specimens were taken by Ondřej Michálek.

**Figure S2** The abundance distribution of adult *Cacopsylla pyricola* (A) and *C. pyrisuga* (B) collected on pears in Starý Lískovec (Brno, Czech Republic = CZ2) throughout an entire year from February 2020 to February 2021. The graphs start in May 2020 for convenience, to show the distribution of generations across different seasons. Black arrows indicate the abundance peaks from which adult psyllid specimens were selected for sequencing.

**Figure S3** Rarefaction curves showing the number of observed ASVs depending on the number of reads at an even sampling depth of 10,000 reads per sample.

**Figure S4** Microbiome composition in individuals of *Cacopsylla pyri*. (A) Including the most abundant taxa *Carsonella*, Group1 *pyri* and *Wolbachia*, and (B) after removing ASVs of *Carsonella*, Group1 *pyri* and *Wolbachia*. Samples with parasitoid DNA (P14–P18) are presented here but were discarded from the analysis.

**Figure S5** Microbiome composition in individuals of *Cacopsylla pyricola*. (A) Including the most abundant taxa *Carsonella* and Group1 *pyricola*, and (B) after removing ASVs of *Carsonella* and Group1 *pyricola*.

**Figure S6** Microbiome composition in individuals of *Cacopsylla pyrisuga*. (**A**) Including the most abundant taxa *Carsonella* and Group2 *pyrisuga*, and (**B**) after removing ASVs of *Carsonella* and Group2 *pyrisuga*.

**Table S1** Metadata for all specimens analysed in this study. Samples with parasitoid DNA (P14–P18) that were discarded from the analyses are highlighted in yellow. Number of ASVs is given after removing ASVs containing <3 reads. Species richness (Chao 1) and diversity (Shannon) indices are based on an even sampling depth of 10,000 reads per sample. For specimens with <10,000 reads, the richness and diversity indices are not defined (ND).

**Table S2** ASV table used for all microbiome analyses. For the specimen ID abbreviations, see Table S1.

**Table S3** Bacterial species richness (Chao 1) and diversity (Shannon Index) in the three *Cacopsylla* species depending on developmental stages, generations (seasonality), localities and gender. Significant *p*‐values are highlighted in bold.

**Table S4** Log CPM (counts per million) values for the 15 bacterial genera identified as either indicator species and/or differentially abundant by IndVal and edgeR, respectively. For information on locality abbreviations, see Table 1.

## Data Availability

All sequences were deposited into Sequence Read Archive (Bioproject PRJNA772591). The Sanger sequence was deposited into GenBank (accession number OK561501).
