# Supplementary figures and images for: Microbiome of pear psyllids: A tale about closely related species sharing their endosymbionts
Source: Environ Microbiol. 2022 Sep 9;24(12):5788–808. doi: 10.1111/1462-2920.16180 (PMC10086859; doi:10.1111/1462-2920.16180)

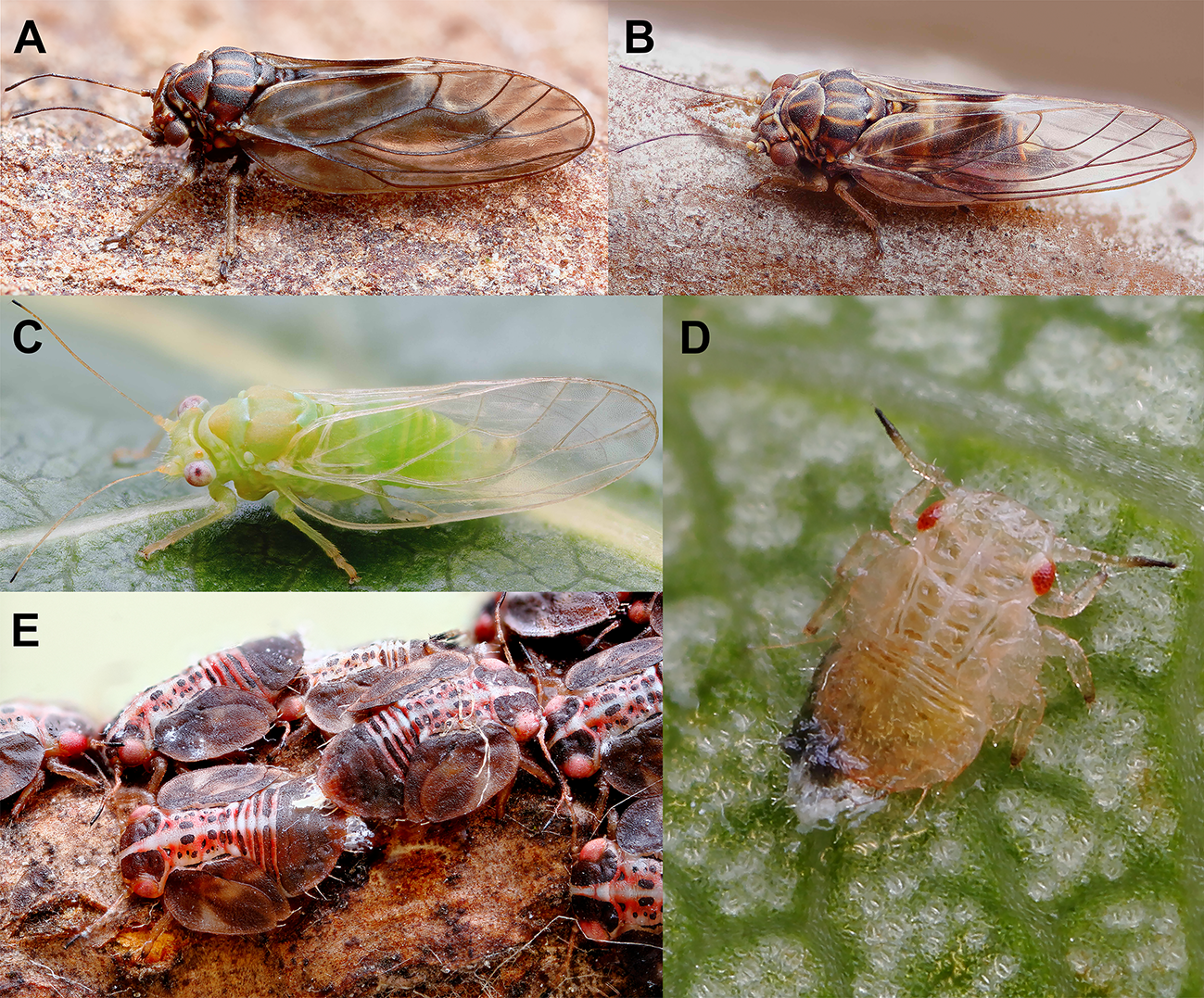

Supplement: Supplementary file 1 — Figure S1 Habitus of adult Cacopsylla pyri – female (A), C. pyricola – female, summer morph (B) and C. pyrisuga – male, freshly emerged, early summer individual (C), and immature C. pyricola (D) and C. pyrisuga (E). The photos from the living specimens were taken by Ondřej Michálek. [file EMI-24-5788-s009.tif]

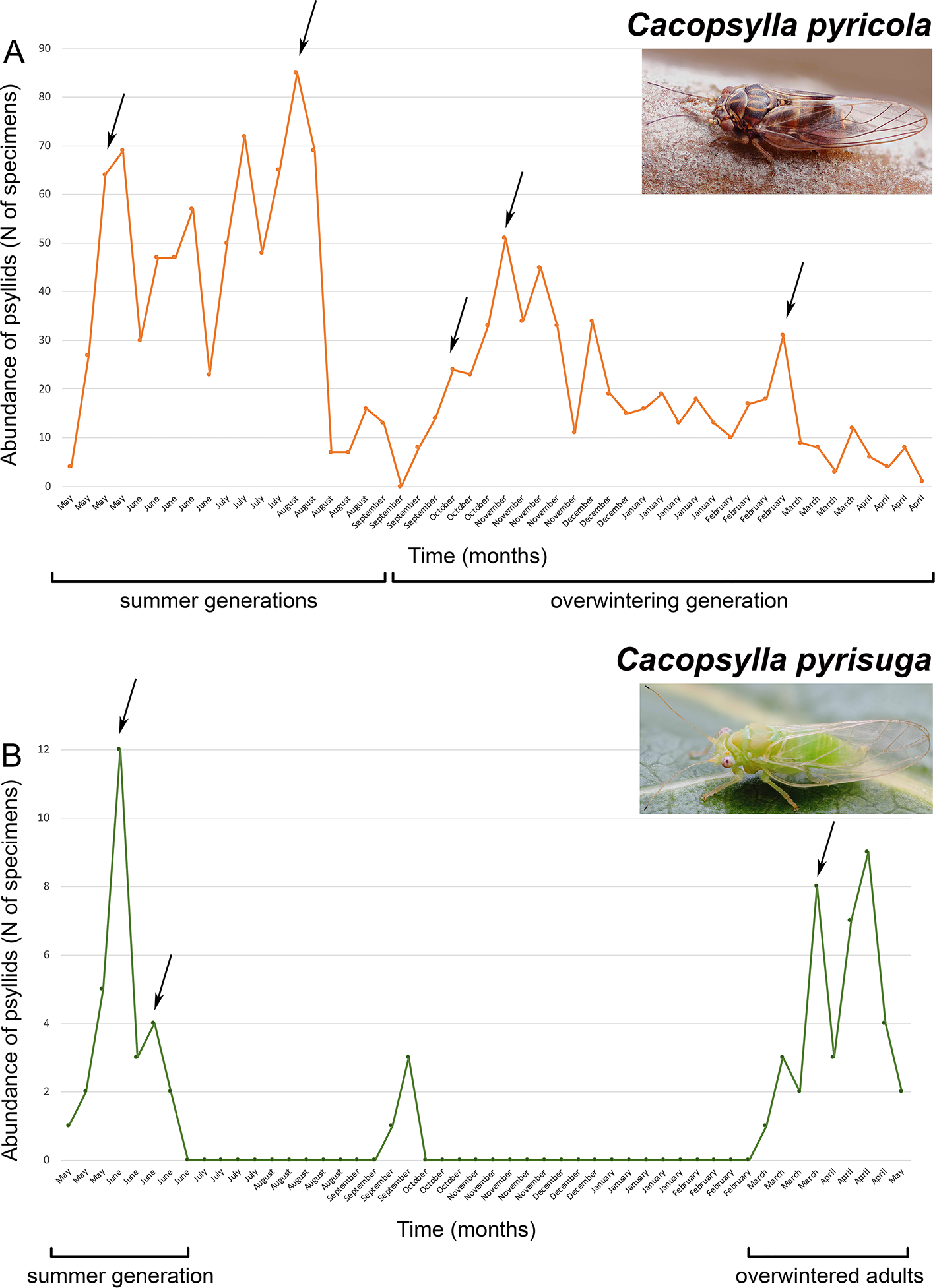

Supplement: Supplementary file 2 — Figure S2 The abundance distribution of adult Cacopsylla pyricola (A) and C. pyrisuga (B) collected on pears in Starý Lískovec (Brno, Czech Republic = CZ2) throughout an entire year from February 2020 to February 2021. The graphs start in May 2020 for convenience, to show the distribution of generations across different seasons. Black arrows indicate the abundance peaks from which adult psyllid specimens were selected for sequencing. [file EMI-24-5788-s007.tif]

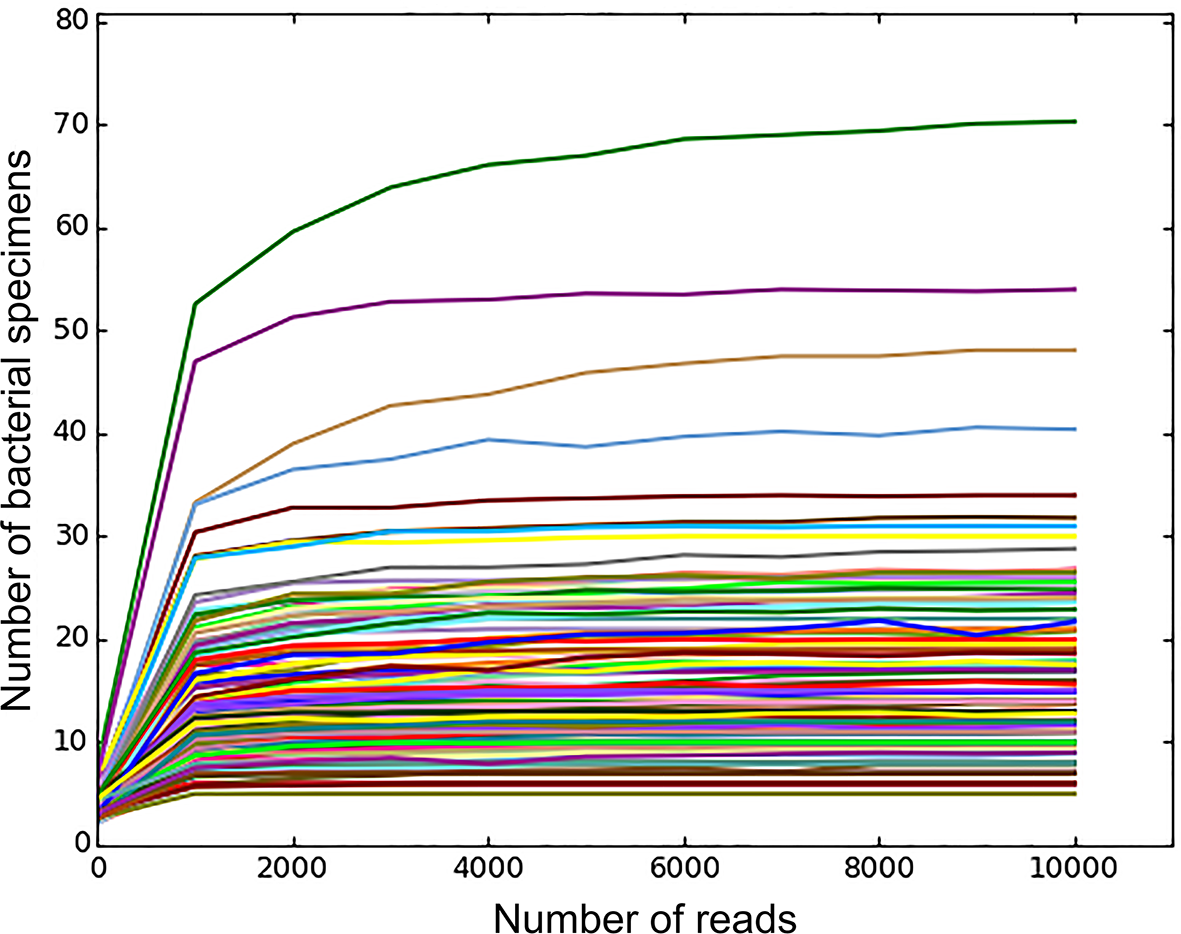

Supplement: Supplementary file 3 — Figure S3 Rarefaction curves showing the number of observed ASVs depending on the number of reads at an even sampling depth of 10,000 reads per sample. [file EMI-24-5788-s003.tif]

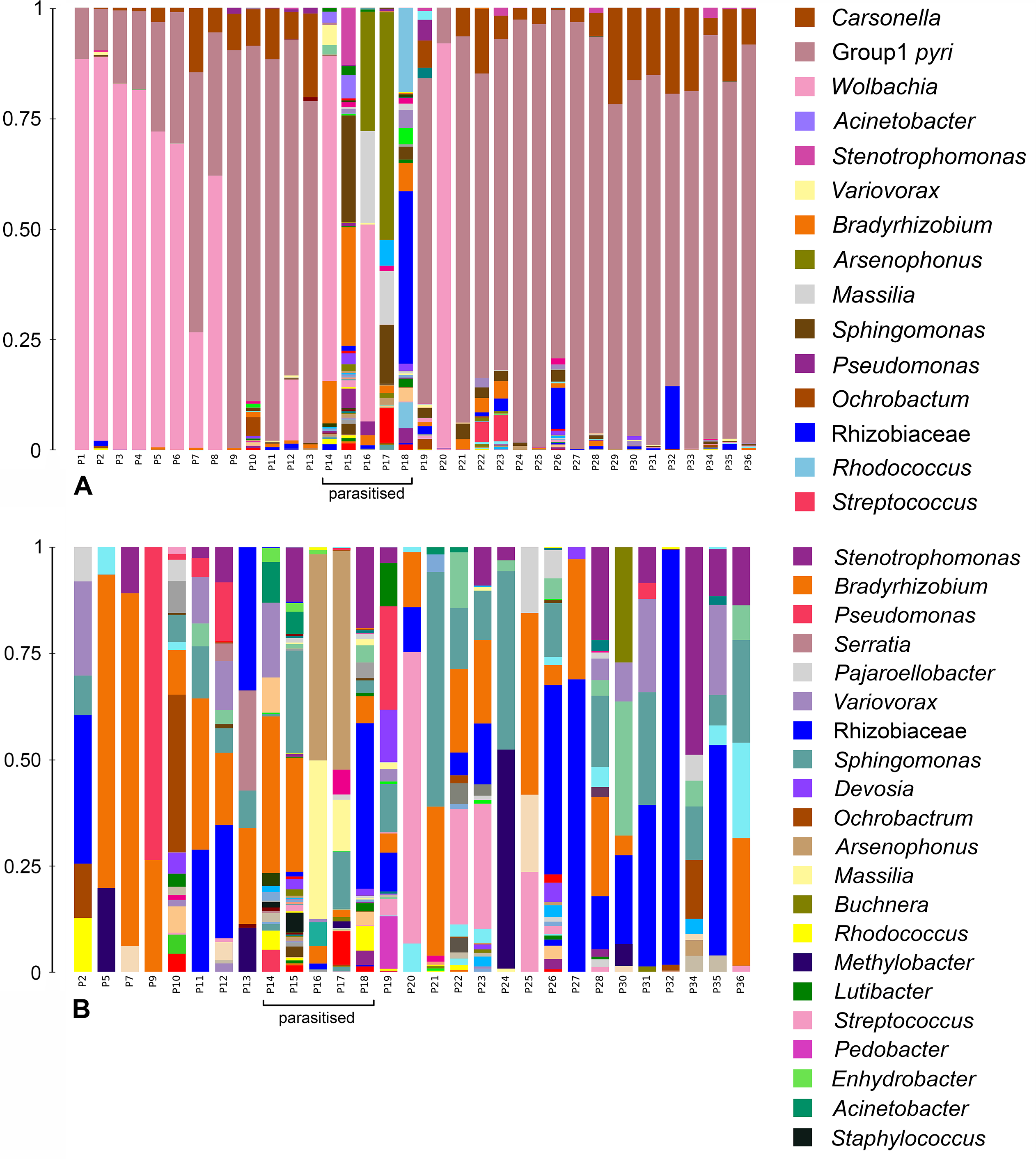

Supplement: Supplementary file 4 — Figure S4 Microbiome composition in individuals of Cacopsylla pyri. (A) Including the most abundant taxa Carsonella, Group1 pyri and Wolbachia, and (B) after removing ASVs of Carsonella, Group1 pyri and Wolbachia. Samples with parasitoid DNA (P14–P18) are presented here but were discarded from the analysis. [file EMI-24-5788-s008.tif]

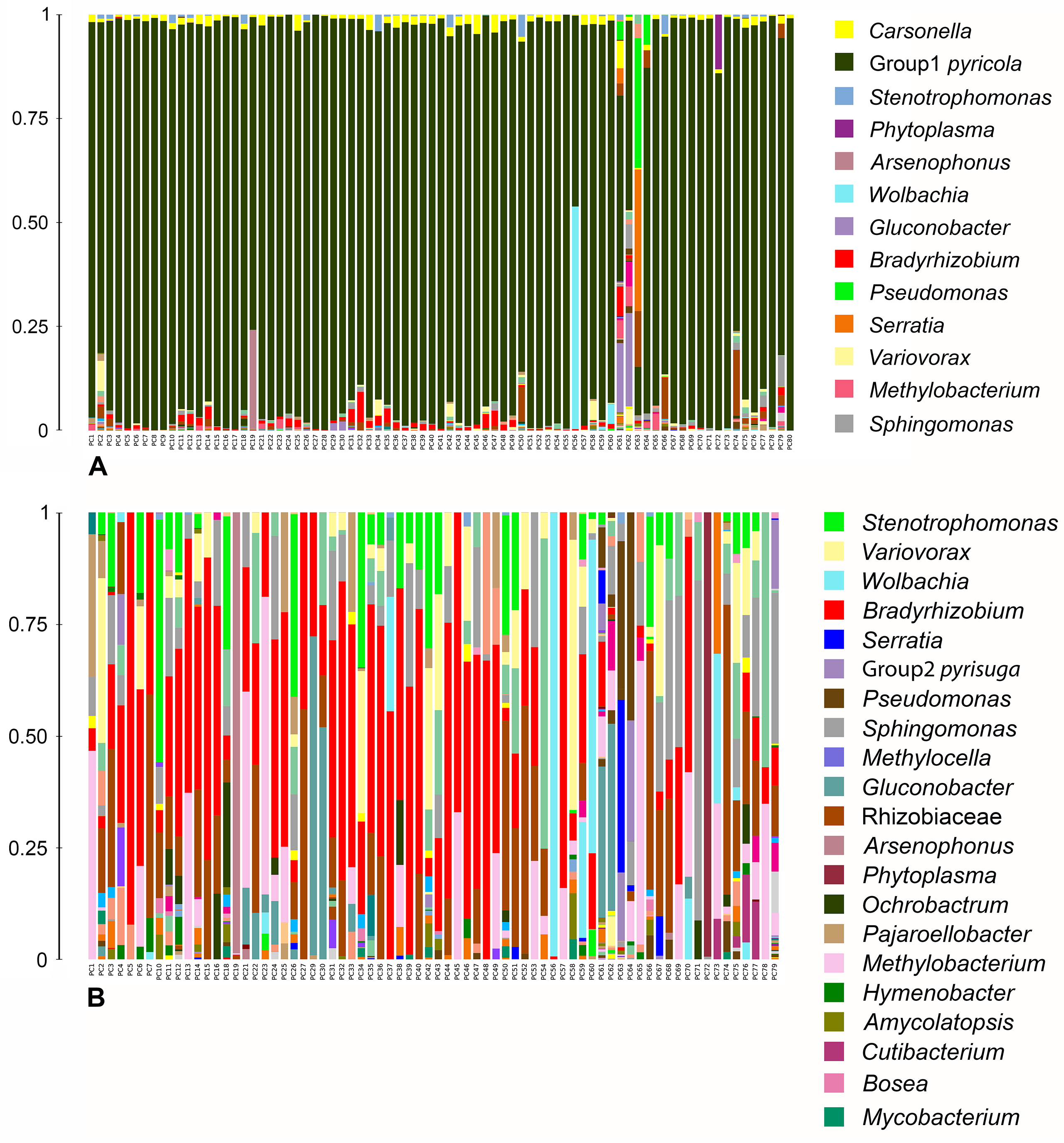

Supplement: Supplementary file 5 — Figure S5 Microbiome composition in individuals of Cacopsylla pyricola. (A) Including the most abundant taxa Carsonella and Group1 pyricola, and (B) after removing ASVs of Carsonella and Group1 pyricola. [file EMI-24-5788-s004.tif]

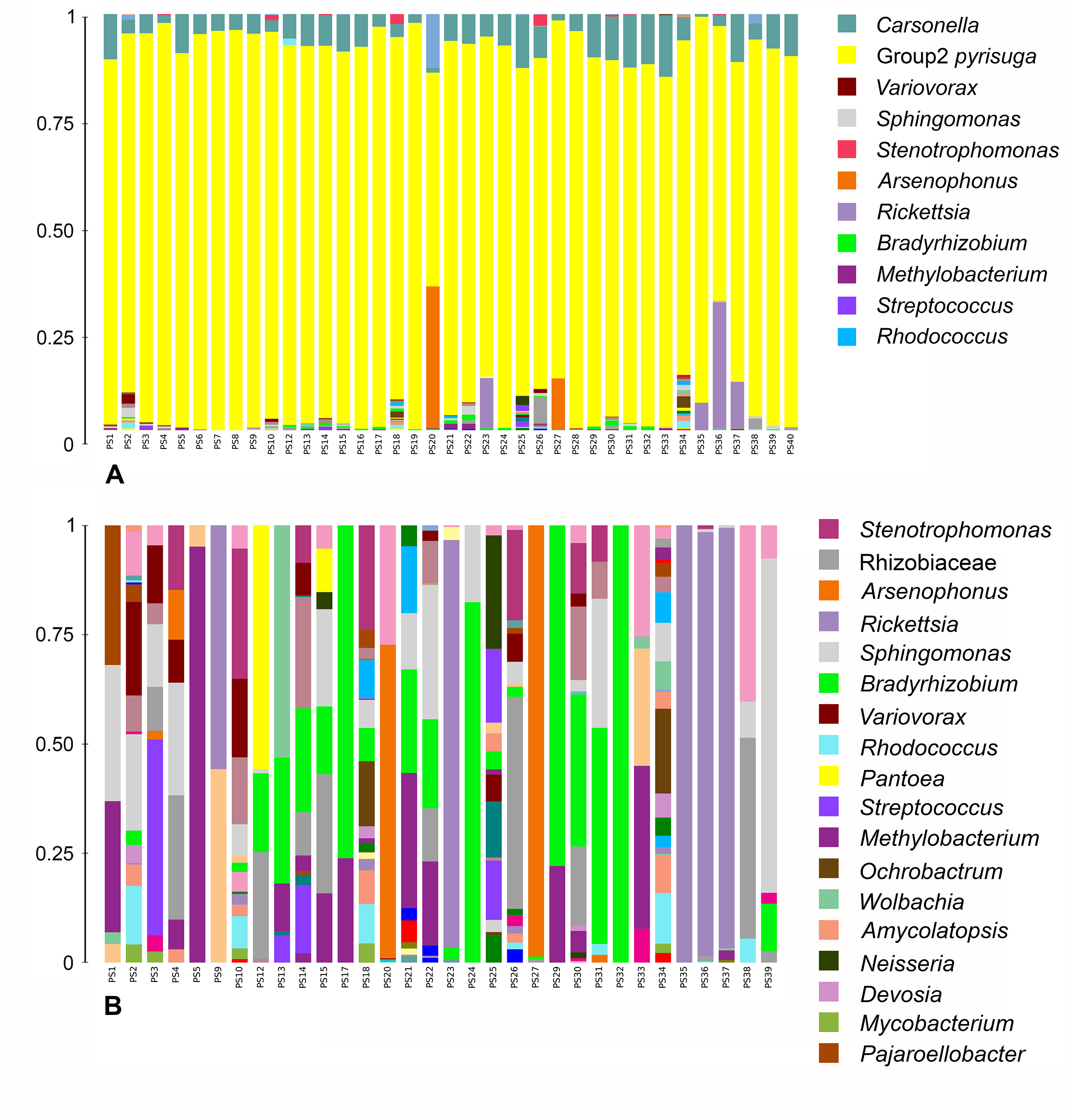

Supplement: Supplementary file 6 — Figure S6 Microbiome composition in individuals of Cacopsylla pyrisuga. (A) Including the most abundant taxa Carsonella and Group2 pyrisuga, and (B) after removing ASVs of Carsonella and Group2 pyrisuga. [file EMI-24-5788-s001.tif]
